# Trifunctional Sialylation‐Based SF‐ZIF@NA Hydrogel for Selective Osteoclast Inhibition and Enhanced Bone‐Vessel Regeneration in Osteoporotic Bone Defects

**DOI:** 10.1002/advs.202415895

**Published:** 2025-03-26

**Authors:** Zhengrong Chen, Wenxin Yang, Yong Tang, Qianqian Dong, Kui Huang, Jiulin Tan, Jie Zhang, Juan Cai, Qixiu Yu, Qijie Dai, Jianzhong Xu, Shuquan Guo, Ce Dou, Fei Luo

**Affiliations:** ^1^ Department of Orthopedics Southwest Hospital Third Military Medical University (Army Medical University) Chongqing 400038 China; ^2^ College of Bioengineering Chongqing University Chongqing 400044 China; ^3^ Integrative Science Center of Germplasm Creation in Western China (CHONGQING) Science City Biological Science Research Center Southwest University Chongqing 400716 China; ^4^ Chongqing Engineering and Technology Research Centerfor Novel Silk Materials Southwest University Chongqing 400715 China; ^5^ Department of Orthopaedics The First Affiliated Hospital of Chongqing Medical University Chongqing 400042 China

**Keywords:** bone regeneration, osteoporosis, pdgf‐bb, sialylation, vascularization

## Abstract

Osteoporotic bone defects are challenging to repair due to imbalances in bone resorption and formation, coupled with insufficient vascularization. To address these issues, it develops a trifunctional hydrogel (SF‐ZIF@NA) designed to selectively inhibit osteoclast activity and enhance vascularized bone regeneration. By enzymatically removing sialic acid, SF‐ZIF@NA prevents precursor osteoclasts (pOCs) from fusing into bone‐resorbing mature osteoclasts (mOCs), thereby preserving pOCs and their anabolic functions. Additionally, the hydrogel releases Zinc ion (Zn^2^⁺) in response to acidic conditions, promoting osteogenesis and angiogenesis. In vitro results confirmed that SF‐ZIF@NA impedes osteoclast fusion, enhances platelet‐derived growth factor‐BB (PDGF‐BB secretion from pOCs, and activates the FAK (focal adhesion kinase) signaling pathway to stimulate vascularized bone formation. In osteoporotic bone defect models, SF‐ZIF@NA accelerated bone repair with increased bone density and vascularization. These findings demonstrate that SF‐ZIF@NA offers a targeted and multifunctional strategy for osteoporotic bone regeneration by concurrently modulating osteoclast activity and promoting angiogenesis.

## Graphical abstract

This study introduces the SF‐ZIF@NA hydrogel system for osteoporotic bone defect treatment. The hydrogel inhibits osteoclast fusion by removing sialic acid and preserves the anabolic function of precursor osteoclasts (pOCs). It also releases Zn^2^⁺ in acidic conditions, promoting osteogenesis and angiogenesis. Together, these effects accelerate the repair of osteoporotic bone defects, improving bone regeneration and vascularization.

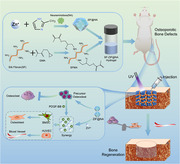

## 1 Introduction

Osteoporotic fractures have become a critical public health issue worldwide, particularly with the aging population, leading to high morbidity, diminished quality of life, and a growing healthcare burden.^[^ ^1^ ^]^ The clinical challenge in treating osteoporotic bone defects lies in simultaneously promoting bone regeneration and ensuring adequate vascularization, both of which are essential for successful bone repair.^[^ ^2^ ^]^ However, excessive bone resorption—characteristic of osteoporosis—further compromises bone integrity and complicates treatment.^[^ ^3^ ^]^ Therefore, effective treatment strategies must achieve a delicate balance between fostering coupled osteogenesis and angiogenesis while suppressing osteoclast‐mediated bone loss.^[^ ^4^ ^]^ These demands underscore the necessity for multifunctional biomaterials that actively modulate the local bone microenvironment, promoting bone formation, vascular integration, and controlled resorption. Developing such biomaterials is essential for advancing osteoporotic fracture treatment and improving patient outcomes.

Osteoclasts, the cells responsible for bone resorption, exist in distinct stages with differing functions: precursor osteoclasts (pOCs) and mature osteoclasts (mOCs).^[^ ^5^ ^]^ pOCs are mononuclear, exhibit minimal resorptive activity, and play an important regulatory and anabolic role in bone biology.^[^ ^6^ ^]^ Unlike mOCs, which drive bone resorption upon multinucleation, pOCs secrete platelet‐derived growth factor‐BB (PDGF‐BB), a key cytokine that promotes the formation of specialized H‐type blood vessels (CD31⁺Emcn⁺), thereby linking angiogenesis with osteogenesis.^[^ ^7^ ^]^ This regulatory function of pOCs is essential for fostering bone regeneration alongside vascularization, making them beneficial cells to preserve. Inhibiting pOC fusion to mOCs presents a promising strategy to simultaneously suppress excessive bone resorption while enhancing bone‐vascular integration. However, existing anti‐resorptive treatments, such as bisphosphonates, lack specificity—they inhibit both pOCs and mOCs, inadvertently suppressing the regenerative potential of pOCs.^[^ ^8^ ^]^ This indiscriminate inhibition compromises long‐term bone healing and increases the risk of atypical fractures. Thus, developing a targeted approach that selectively depletes mOCs while preserving pOCs could provide a superior therapeutic strategy for osteoporotic bone defects.

To selectively deplete mOCs while preserving pOCs, we introduce an innovative approach that targets sialic acids on osteoclast surfaces. We previously showed that pOCs recognize and fuse with each other through Siglec‐15, a sialic acid‐binding immunoglobulin‐like lectin, interacting with sialylated Toll‐like receptor 2 (TLR2).^[^ ^9^ ^]^ In this study, we leveraged neuraminidase (NA) to enzymatically remove sialic acids, disrupting this fusion process and effectively preventing pOCs from maturing into resorptive mOCs while maintaining their anabolic function. This selective inhibition of mOC formation allows pOCs to remain active, secreting PDGF‐BB and supporting bone‐vessel regeneration. Notably, this sialic acid‐targeting approach represents a novel and precise strategy to simultaneously regulate osteoclast activity and optimize bone repair, addressing a major challenge in osteoporosis treatment.

**Scheme 1:**
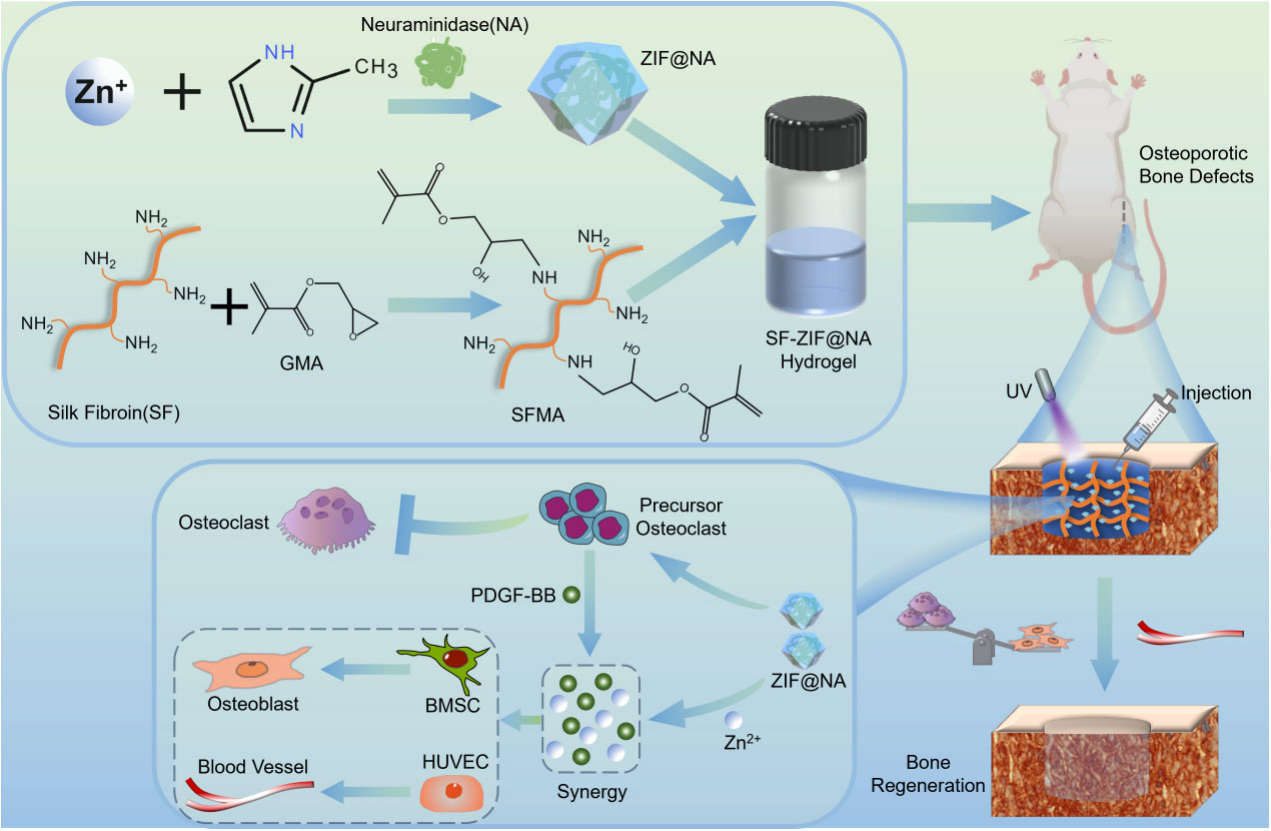
Schematic illustration of the construction and application of the SF‐ZIF@NA hydrogel system for the treatment of osteoporotic bone defects.

This study introduces a sialylation‐targeting hydrogel (SF‐ZIF@NA) as a multifunctional biomaterial to modulate osteoporotic bone repair. Through sialic acid removal and pH‐responsive Zinc ion (Zn^2^⁺) release, SF‐ZIF@NA promotes both osteogenesis and angiogenesis within the acidic osteoporotic microenvironment (**Scheme** **1**). We hypothesize that this trifunctional strategy provides a targeted, effective solution for accelerating vascularized bone regeneration while mitigating excessive bone resorption, thereby addressing a critical gap in osteoporotic treatment.

## 2 Results and Discussion

### 2.1 Characterization and Enzymatic Activity Assessment of ZIF@NA

**Figure 1:**
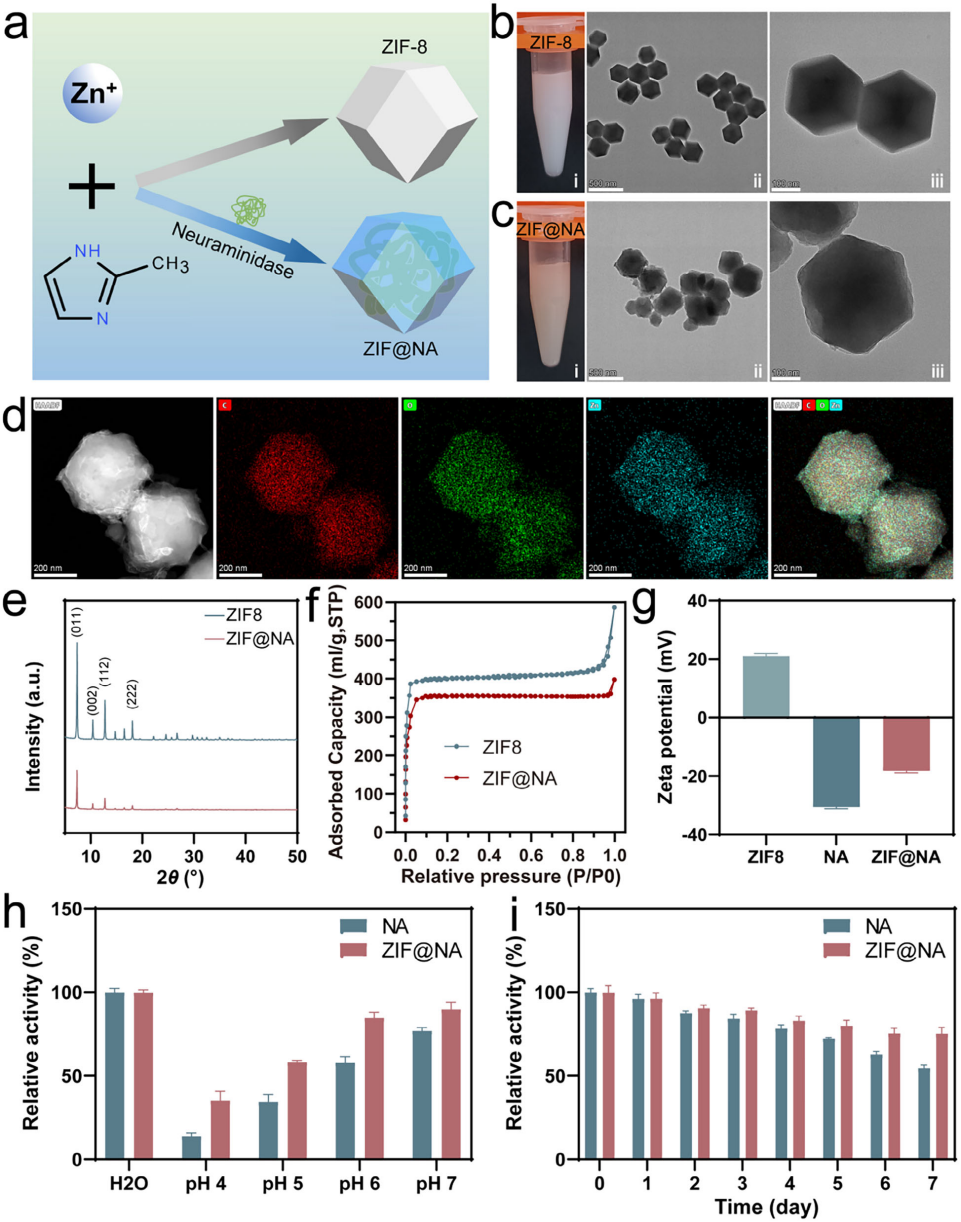
Characterization of ZIF@NA. a) Schematic diagram of ZIF8 and ZIF@NA preparation; TEM images of b) ZIF8 and c) ZIF@NA (ii scale bar: 500 nm; iii scale bar: 100 nm); d) Corresponding elemental mappings of C, O and Zn in ZIF@NA (scale bar: 200 nm); e) XRD patterns of ZIF8 and ZIF@NA; f) Nitrogen adsorption of ZIF8 and ZIF@NA; g) Zeta potential of ZIF8, NA and ZIF@NA (n = 3); h) Relative enzymatic activities of NA and ZIF@NA after different PH treatments (n = 3); i) Relative enzymatic activities of NA and ZIF@NA after treatment at different times (n = 3). Data presented as mean ± standard deviation (SD).

Zeolitic imidazolate framework‐8 (ZIF‐8), formed by linking Zn^2^⁺ nodes and HmIM through robust Zn@N bonds, is highly conducive to enzyme encapsulation due to its biocompatible synthesis conditions and relatively low cytotoxicity.^[^ ^10^ ^]^ NA was encapsulated within ZIF‐8, generating the composite ZIF@NA, as illustrated in Figure **1**a. The enzyme loading capacity was determined to be ≈25.88 wt.%, based on the enzymatic activity analysis of the supernatant before and after encapsulation (Figure S1 and Table S1, Supporting Information). The resulting ZIF@NA dispersion exhibited a pale yellow hue in contrast to the white dispersion of pristine ZIF‐8 (Figure 1b,c). Transmission electron microscopy (TEM) images confirmed that ZIF‐8 exhibited the expected dodecahedral morphology (Figure 1b). However, after NA incorporation, ZIF@NA adopted an irregular polyhedral structure (Figure 1c). This morphological shift is likely due to NA acting as nucleation seeds, influencing the crystallization dynamics of ZIF‐8 and altering its final shape.^[^ ^11^ ^]^ Elemental mapping further verified the uniform distribution of carbon (C), oxygen (O), and zinc (Zn) throughout the ZIF@NA composite (Figure 1d). Particle size distribution analysis (Figure S2, Supporting Information) revealed no substantial differences between ZIF‐8 and ZIF@NA, with both displaying sizes in the 300–600 nm range. The slightly larger size observed compared to TEM results likely arises from minor nanoparticle aggregation in suspension, a common occurrence in aqueous dispersions.^[^ ^12^ ^]^ X‐ray diffraction (XRD) analysis (Figure 1e) revealed the characteristic diffraction peaks of ZIF‐8 at 7.3°, 10.4°, 12.7°, and 18.0°, corresponding to the (011), (002), (112), and (222) planes, respectively, consistent with standard ZIF‐8 diffraction patterns. The retention of these peaks in ZIF@NA indicates that the crystalline integrity of ZIF‐8 was largely preserved post‐encapsulation. However, the intensity of the peaks slightly diminished, likely due to the presence of amorphous NA, suggesting a partial reduction in crystallinity. As shown in Figure 1f, both ZIF8 and ZIF@NA exhibited typical type I N₂ adsorption‐desorption isotherms, which are characteristic of microporous materials. The Brunauer‐emmett‐teller (BET) surface area of ZIF8 was calculated to be 1168.91 m^2^ g^−^¹, while the incorporation of NA reduced the BET surface area of ZIF@NA to 1059.54 m^2^ g^−^¹. Additionally, the corresponding pore volumes of ZIF8 and ZIF@NA were 0.84 cm^3^ g^−^¹ and 0.58 cm^3^ g^−^¹, respectively. This decline in both BET surface area and pore volume reflects the successful loading of NA within the ZIF8 framework, effectively occupying its internal pore spaces. Zeta potential analysis (Figure 1g) provided strong evidence of the successful encapsulation of NA within the ZIF‐8 framework. ZIF‐8 demonstrated a positive zeta potential of 20.97 mV, primarily due to the presence of Zn^2^⁺, reflecting its positively charged surface. Conversely, NA exhibited a negative zeta potential of −30.60 mV, consistent with the enzyme's negatively charged surface. During the crystallization process, the electrostatic interactions between the positively charged Zn^2^⁺ in ZIF‐8 and the negatively charged NA molecules facilitated the efficient encapsulation of NA within the ZIF‐8 matrix.^[^ ^13^ ^]^ After encapsulation, the zeta potential of ZIF@NA shifted to −18.23 mV, which, being lower than ZIF‐8 but higher than NA alone, suggests partial neutralization of charges, further corroborating the successful formation of the ZIF@NA composite. To confirm the encapsulation of NA, Cy5.5‐labeled NA (with red fluorescence) was employed and visualized under confocal microscopy. The appearance of red fluorescence in ZIF@NA (Figure S3, Supporting Information) confirmed the successful incorporation of NA into the ZIF‐8 structure.

The bioactivity of the encapsulated enzyme was assessed under varying pH conditions to evaluate the protective effect of ZIF‐8. As depicted in Figure 1h, both free NA and ZIF@NA exhibited reduced activity under acidic conditions. However, the relative activity of ZIF@NA was significantly higher than that of the free enzyme. This enhanced stability can be attributed to the protective role of the ZIF‐8 matrix, which likely shields the enzyme's structure from proton‐mediated degradation in acidic environments, thus reducing the risk of denaturation or inactivation.^[^ ^14^ ^]^ In addition, long‐term storage stability of ZIF@NA was evaluated over seven days (Figure 1i). While the activity of free NA progressively decreased over time, with a marked decline between days 5 and 7, ZIF@NA exhibited only a slight reduction in activity and maintained a more stable performance overall. These results demonstrate that ZIF encapsulation not only improves enzyme tolerance to acidic environments but also enhances the enzyme's retention of activity during extended storage and use. In the context of osteoporosis, where osteoclasts secrete acidic substances and hydrolytic enzymes that exacerbate the local acidic environment, ZIF‐8 encapsulation of neuraminidase could offer a significant therapeutic advantage.^[^ ^15^ ^]^ By maintaining greater stability and activity in acidic microenvironments, ZIF@NA could extend the functional lifespan of the enzyme, potentially improving therapeutic outcomes in conditions characterized by acidic microenvironments.

### 2.2 Characterization and Biocompatibility of SF‐ZIF@NA Hydrogel

**Figure 2:**
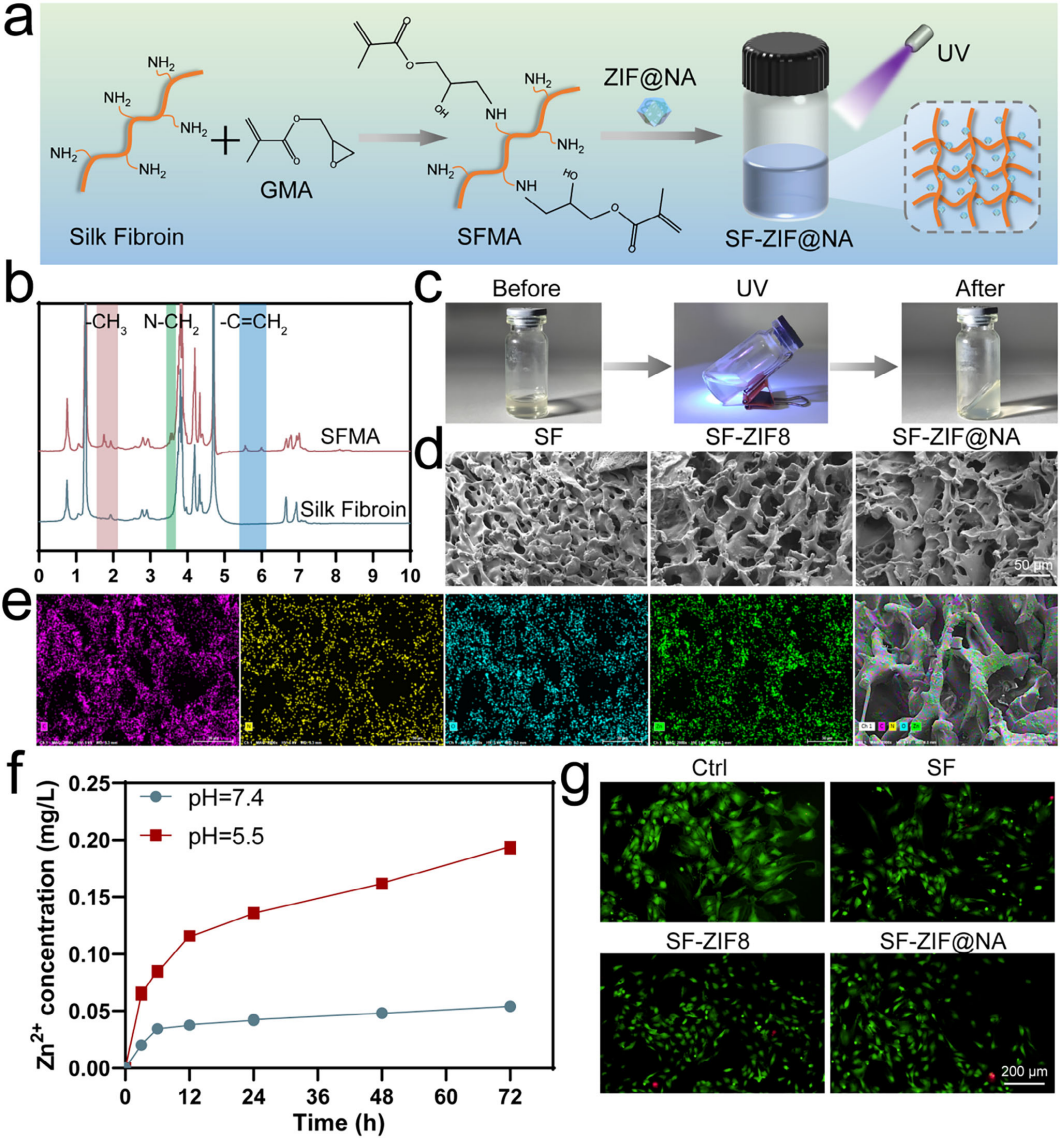
Preparation and characterization of SF‐ZIF@NA hydrogel. a) Schematic diagram of the preparation of SF‐ZIF@NA hydrogel; b) ^1^H NMR spectra of unsubstituted SF and SFMA; c) UV crosslinking process of the SF‐ZIF@NA hydrogel; d) SEM images of SF, SF‐ZIF8, and SF‐ZIF@NA; e) Corresponding elemental mappings of C, N, O, and Zn in SF‐ZIF@NA; f); g) Fluorescence microscopy images of live/dead detection of BMSC incubated in well plates or co‐cultured on hydrogels for 24 h.

The direct incorporation of nanoparticles into bone defect sites often leads to poor retention and inconsistent therapeutic outcomes, mainly due to their diffusion and instability in vivo.^[^ ^16^ ^]^ Silk fibroin hydrogels address these challenges by providing a mechanically supportive scaffold that stabilizes nanoparticle dispersion and enables their sustained release. As shown in **Figure** **2**a, the preparation of SF‐ZIF@NA hydrogel involves chemically modifying silk fibroin by grafting methacryloyl (GMA) groups to produce SFMA. This modified SFMA is then combined with ZIF@NA, followed by UV‐induced crosslinking to form the SF‐ZIF@NA hydrogel. Figure 2b presents the ¹H NMR spectra of both unmodified silk fibroin and SFMA. In the SFMA spectrum, new peaks observed at ≈5.5 ppm and 6.1 ppm, corresponding to the vinyl double bonds (‐C = CH₂), confirm the successful introduction of methacrylate groups.^[^ ^17^ ^]^ This modification is critical for facilitating the subsequent photo‐crosslinking process, enhancing the mechanical stability and durability of the hydrogel (Figure 2c). To evaluate the mechanical properties of the hydrogel, stress‐strain analysis and elastic modulus measurements were performed (Figure S4, Supporting Information). The results showed that the incorporation of ZIF@NA enhanced the hydrogel's elastic modulus, reaching ≈0.52 MPa, higher than that of pure SF hydrogel and comparable to the SF‐ZIF8 hydrogel. This improvement is attributed to the energy dissipation facilitated by strong interfacial interactions between the nanoparticles and the hydrogel matrix.^[^ ^18^ ^]^ Additionally, the hydrogel's injectability and in situ crosslinking capability (Movie S1, Supporting Information) enable it to conform to irregular defect sites while maintaining structural stability post‐crosslinking. Scanning electron microscopy (SEM) images (Figure 2d; Figure S5, Supporting Information) reveal the morphological features of SF, SF‐ZIF8, and SF‐ZIF@NA hydrogels. Pure silk fibroin shows a porous structure, which becomes more defined upon the incorporation of ZIF8, and this porous network is retained in the SF‐ZIF@NA hydrogel. The uniform dispersion of nanoparticles within the hydrogel matrix ensures that the porous structure is maintained, which is essential for promoting nutrient transport and cell infiltration in bone tissue engineering.^[^ ^19^ ^]^ Moreover, elemental mapping of SF‐ZIF@NA (Figure 2e) confirms a homogeneous distribution of C, nitrogen (N), O, and Zn throughout the hydrogel matrix. This consistent dispersion of ZIF@NA nanoparticles is critical for maintaining uniform functional performance throughout the hydrogel. Figure 2f demonstrates the Zn^2^⁺ release profile from SF‐ZIF@NA hydrogels under different pH conditions. The release rate of Zn^2^⁺ increases significantly under acidic conditions (pH 5.5), mimicking the microenvironment of osteoporotic bone defects, while remaining relatively stable at neutral pH (7.4), representing physiological conditions. This controlled release behavior ensures that therapeutic ions are delivered more effectively in response to the local environment, optimizing the healing process without excessive release in healthy tissues.^[^ ^20^ ^]^ The degradation behavior of the hydrogels followed a similar pH‐dependent trend (Figure S6, Supporting Information). At pH 5.5, the degradation rate increased more rapidly, reaching ≈35% by day 14, compared to ≈25% at pH 7.4. This difference is likely due to the enhanced hydrolysis of silk fibroin and destabilization of the ZIF framework in acidic conditions. The faster degradation in acidic environments complements the increased Zn^2^⁺ release, promoting ion delivery in pathological conditions. Meanwhile, the slower degradation under neutral pH ensures prolonged structural stability and sustained therapeutic effects.

Biocompatibility is crucial for the application of SF‐ZIF@NA hydrogels in osteoporotic bone defect repair. The effect of different ZIF@NA concentrations on the viability of bone marrow stromal cells (BMSCs) and RAW macrophages was evaluated (Figures S7 and S8, Supporting Information). A dose‐dependent response was observed in both cell types. At concentrations ≤1 mg mL^−1^, cell viability remained high and comparable to the control group, indicating minimal cytotoxicity. However, at 2 mg mL^−1^, a significant reduction in cell viability was noted, with BMSC and RAW viability decreasing to ≈60%. This suggests that higher concentrations of ZIF@NA may induce cytotoxic effects. Given that a relative cell viability of 70% or higher is generally considered acceptable for biocompatibility, a concentration of 1 mg mL^−1^ was identified as optimal for further experiments.^[^ ^21^ ^]^ The Cell counting kit‐8 (CCK‐8) assay results (Figure S9, Supporting Information) show the proliferation of BMSCs co‐cultured with the hydrogels over 1, 3, and 7 days. No significant differences in cell proliferation were observed among the SF, SF‐ZIF8, and SF‐ZIF@NA groups compared to the control, indicating that the addition of ZIF@NA does not negatively impact cell viability. This level of biocompatibility, comparable to that of SF and SF‐ZIF8 hydrogels, affirms the potential of SF‐ZIF@NA for bone tissue engineering applications. Additionally, the live/dead assay (Figure 2g) provides further evidence of the hydrogels' biocompatibility. Fluorescence microscopy images show a high proportion of live cells (green fluorescence) with minimal dead cells (red fluorescence) after 24 h of co‐culture with BMSCs. Notably, the SF‐ZIF@NA hydrogel supports cell survival and exhibits similar viability to the control, SF, and SF‐ZIF8 hydrogels (Figure S10, Supporting Information). This demonstrates that the incorporation of ZIF8 and NA does not compromise cell survival, and the hydrogel provides a favorable microenvironment for cell growth.

### 2.3 Effects of SF‐ZIF@NA Hydrogel on Osteoclast

**Figure 3:**
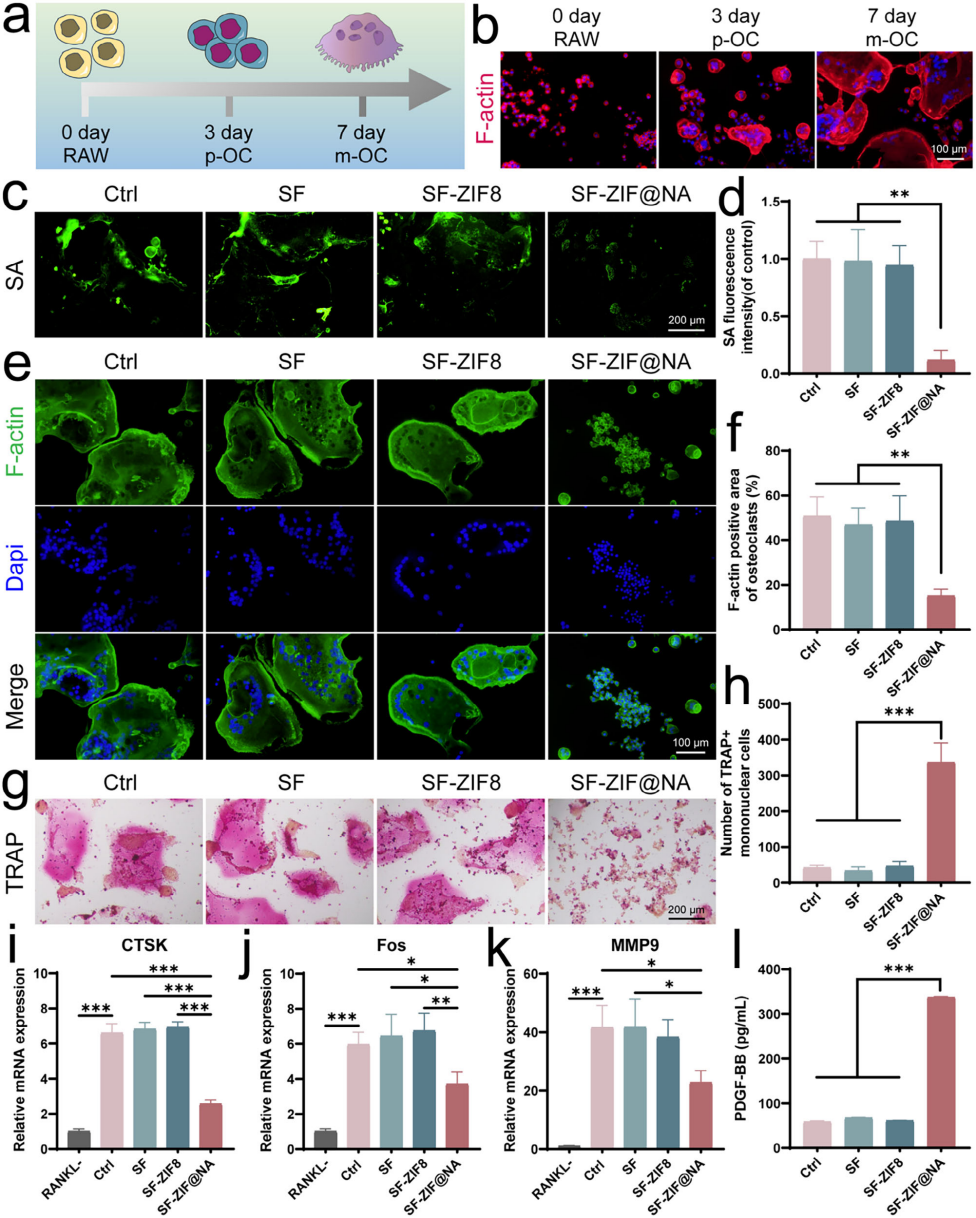
Effect of the hydrogel on osteoclastogenic differentiation of RAW264.7. a) Schematic and b) fluorescence image of osteoclast fusion process in the presence of RANKL; c) Sialic acid staining images and d) quantitative analysis of induced cells at 3 days (n = 3, one‐way ANOVA); e) The nuclear/F‐actin staining of induced cells at 7 days; f) Quantitative analysis of osteoclast area in induced cells at 7 days (n = 3, one‐way ANOVA); g) TRAP staining of induced cells at 7 days; h) Quantitative analysis of the number of TRAP‐positive mononuclear cells (n = 3, one‐way ANOVA); i‐k) The relative mRNA expression levels of osteoclast‐related markers CTSK, Fos, and MMP9 after 7 days of culture (n = 3, one‐way ANOVA); l) Detection of PDGF‐BB in the conditioned medium of induced cells after 7 days using ELISA (n = 3, one‐way ANOVA). Data are presented as mean ± SD. *p < 0.05, **p < 0.01, ***p < 0.001 indicate statistical significance.

We next investigated the effects of SF‐ZIF@NA on osteoclastogenesis and the expression of osteoclast‐related genes. In the presence of receptor activator of nuclear factor kappa‐B ligand (RANKL), mononuclear macrophages proliferate and differentiate, eventually fusing into multinucleated, mature osteoclasts—the primary contributors of bone resorption in osteoporotic conditions (**Figure** **3**a,b). We previously showed that pOCs recognize each other through binding of Siglec‐15, a sialic acid‐binding immunoglobulin‐like lectin, with sialylated TLR2, facilitating the fusion of pOCs into mature, multinucleated mOCs.^[^ ^9^ ^]^ Considering the crucial role of sialic acid in osteoclast precursor fusion, we first examined the impact of SF‐ZIF@NA on sialic acid clearance. As shown in Figure 3c,d, sialic acid is highly expressed during the fusion process, but its expression was significantly reduced in the SF‐ZIF@NA‐treated group compared to other groups. By targeting sialic acid at an early stage of osteoclastogenesis, SF‐ZIF@NA disrupts the precursor fusion process, as evidenced by subsequent alterations in osteoclast morphology and function. Nuclear and filamentous actin (F‐actin) staining at day 7 (Figure 3e) revealed fewer large, multinucleated osteoclasts in the SF‐ZIF@NA group, with reduced formation of F‐actin rings—structures critical for osteoclast attachment and bone resorption.^[^ ^22^ ^]^ This observation is further supported by a quantitative reduction in osteoclast area (Figure 3f), indicating that the majority of cells in the SF‐ZIF@NA group did not progress into fully differentiated, bone‐resorbing osteoclasts. In contrast, the SF‐ZIF8 group did not show a noticeable suppression of osteoclast formation or F‐actin ring development compared to the Ctrl group. While Zn^2+^ are known to suppress osteoclastogenesis, studies have shown that only at sufficiently high concentrations can they effectively interfere with RANKL‐induced signaling pathways.^[^ ^23^ ^]^ In the SF‐ZIF8 group, the release of Zn^2+^ likely remained below the critical threshold required for substantial inhibition, thereby failing to disrupt osteoclast differentiation and function. The effects of SF‐ZIF@NA hydrogel on the bone‐resorptive activity of osteoclasts were further evaluated using a pit formation assay on bovine bone slices (Figure S11, Supporting Information). In the ctrl group, extensive resorption pits were observed, indicating active osteoclastic bone resorption. In contrast, the SF‐ZIF@NA group exhibited markedly reduced resorption pit areas, with only sparse and shallow pits visible on the bone surface. This result indicates that SF‐ZIF@NA effectively suppresses the bone‐resorptive activity of osteoclasts.

At the molecular level, PCR analysis confirmed the inhibitory effects of SF‐ZIF@NA on the transcription of osteoclast‐related genes. As shown in Figure 3i‐k, the mRNA expression levels of CTSK, Fos, and MMP9 were significantly reduced in the SF‐ZIF@NA‐treated group compared to the other groups. The significant reduction in CTSK expression reflects a decrease in cathepsin K, a protease essential for bone matrix degradation.^[^ ^24^ ^]^ Meanwhile, the suppression of Fos disrupts transcriptional regulation necessary for osteoclast differentiation, and the marked decrease in MMP9, a matrix metalloproteinase involved in extracellular matrix breakdown, further corroborates SF‐ZIF@NA's capacity to inhibit essential molecular pathways required for osteoclast maturation and activity.^[^ ^25^ ^]^ Western blot analysis provided further evidence of SF‐ZIF@NA's inhibitory effects at the protein level. As shown in Figure S12 (Supporting Information), the protein levels of CTSK, Fos, and NFATc1 were markedly reduced in the SF‐ZIF@NA group compared to the other groups. These molecular alterations align with the morphological and functional data presented earlier, reinforcing the conclusion that SF‐ZIF@NA effectively suppresses osteoclastogenesis at both cellular and molecular levels.

Interestingly, SF‐ZIF@NA treatment also preserved a population of pOCs, as reflected by a significant increase in their number by day 7 (Figure 3g,h). While these cells express tartrate‐resistant acid phosphatase (TRAP), they do not fuse into multinucleated osteoclasts, thereby preserving their regenerative potential. Notably, these pOCs secrete PDGF‐BB, a key factor for osteogenesis and angiogenesis. ELISA results (Figure 3l) show a significant increase in PDGF‐BB secretion in the SF‐ZIF@NA‐treated group at day 7, suggesting that SF‐ZIF@NA promotes the maintenance of a pro‐regenerative cell population. This dual action—preventing excessive osteoclast fusion while preserving pOCs that continue to secrete PDGF‐BB—highlights the therapeutic potential of SF‐ZIF@NA in treating osteoporotic bone defects.

### 2.4 Effects of SF‐ZIF@NA Hydrogel on Osteogenic Differentiation Through Preserving pOCs

**Figure 4:**
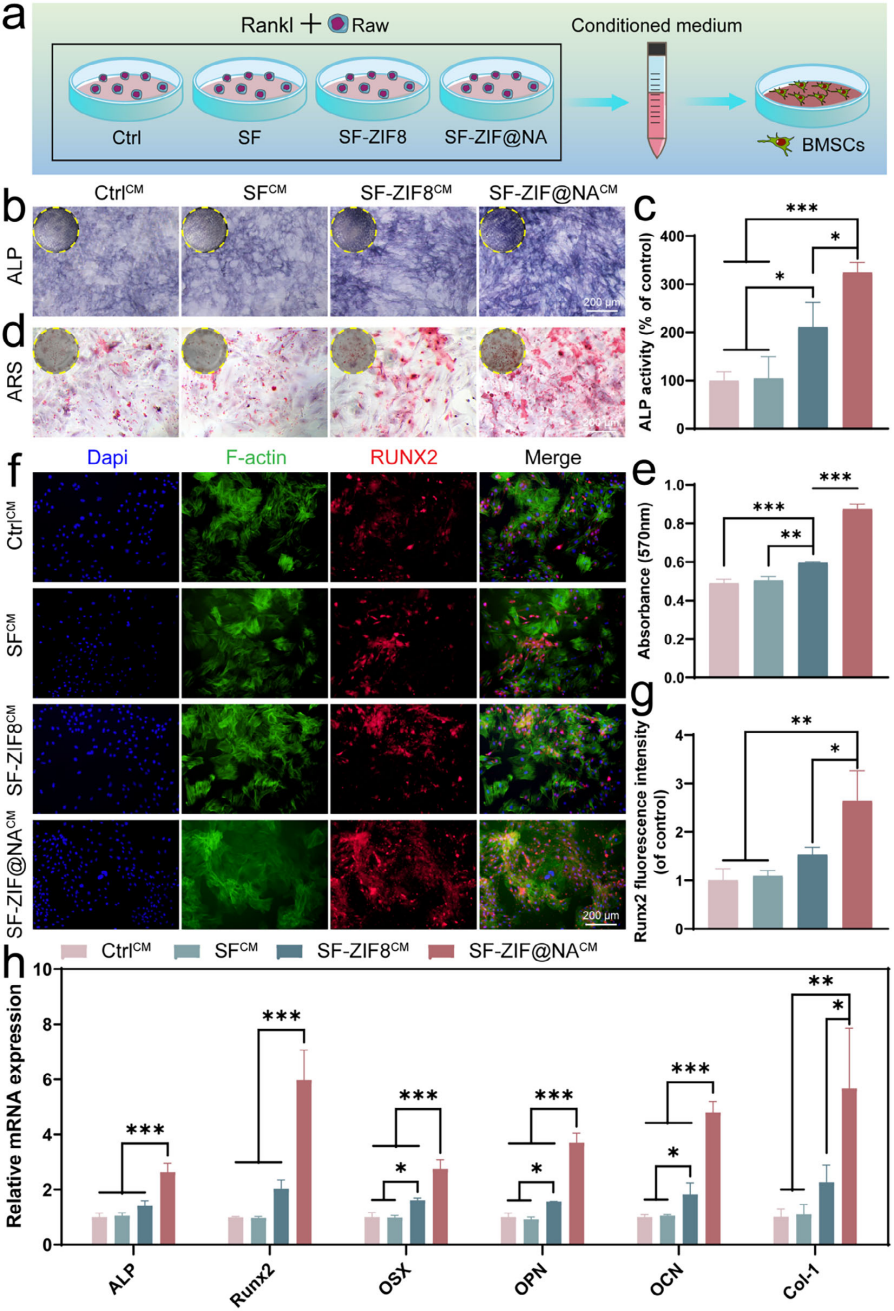
SF‐ZIF@NA preserved pOCs induce osteogenesis. a) Diagram exploring the effects of conditioned medium collected from RANKL‐induced macrophages treated with different hydrogels on BMSC osteogenic differentiation; b) ALP staining images and c) quantitative analysis of induced BMSCs at 7 days (n = 3, one‐way ANOVA); d) ARS staining images and e) quantitative analysis of induced BMSCs at 14 days (n = 3, one‐way ANOVA); f) The nuclear/F‐actin/Runx2 staining of induced BMSCs at 7 days; g) Quantitative analysis of the relative fluorescence intensity of RUNX2 (n = 3, one‐way ANOVA); h) The relative mRNA expression levels of Osteogenesis‐related markers ALP, Runx2, OSX, OPN, OCN, and Col‐1 after 7 days of culture (n = 3, one‐way ANOVA). Data are presented as mean ± SD. *p < 0.05, **p < 0.01, ***p < 0.001 indicate statistical significance.

Since pOCs are beneficial for bone formation, mineralization, and angiogenesis through coupling with osteoblasts and secreting PDGF‐BB.^[^ ^26^ ^]^ To evaluate the effect of SF‐ZIF@NA‐treated osteoclasts on osteogenesis, we cultured BMSCs with conditioned medium collected from RANKL‐induced RAW264.7 treated with different hydrogels (**Figure** **4**a). At day 7, alkaline phosphatase (ALP) staining and quantitative analysis (Figure 4b,c) revealed a significant increase in ALP activity in both SF‐ZIF8^CM^ and SF‐ZIF@NA^CM^ groups compared to the Ctrl^CM^ group. This enhancement in ALP activity is indicative of early osteogenic differentiation. The release of Zn^2+^ from ZIF‐8 in both hydrogels plays a supportive role in promoting osteogenic differentiation. Notably, the SF‐ZIF@NA^CM^ group exhibited the highest ALP activity, surpassing the SF‐ZIF8^CM^ group. This can be attributed to the action of NA in SF‐ZIF@NA, which removes sialic acid, inhibits osteoclast fusion, and maintains the secretion of pro‐regenerative factors such as PDGF‐BB from pOCs. These factors further stimulate BMSC early differentiation, creating a synergistic environment with the Zn^2+^ to promote osteogenesis. By day 14, the alizarin red staining (ARS) results and quantitative analysis (Figure 4d,e) show a significant increase in calcium deposition in both the SF‐ZIF8^CM^ and SF‐ZIF@NA^CM^ groups compared to the Ctrl^CM^ group. The SF‐ZIF@NA^CM^ group demonstrates the highest level of calcium deposition, indicating superior matrix mineralization. The enhanced mineralization observed in the SF‐ZIF@NA^CM^ group is likely due to the combined effects of Zn^2+^ release and the secretion of growth factors, which create a more favorable environment for extracellular matrix maturation and mineral deposition.

Runt‐related transcription factor 2 (Runx2), a pivotal transcription factor regulating osteogenic differentiation,^[^ ^27^ ^]^ was significantly upregulated in both the SF‐ZIF8^CM^ and SF‐ZIF@NA^CM^ groups by day 7 (Figure 4f,g). The elevated expression of Runx2 in the SF‐ZIF@NA^CM^ group underscores the enhanced osteoinductive environment created by the synergistic effects of growth factors secreted by pOCs and the presence of Zn^2+^. This is reflected in the elevated expression of Runx2 and other osteogenesis‐related genes in the SF‐ZIF@NA^CM^ group, as shown in the mRNA analysis (Figure 4h). All osteogenic markers, including ALP, Runx2, Osterix (OSX), Osteopontin (OPN), Osteocalcin (OCN), and Collagen Type 1 (Col‐1), are significantly upregulated in SF‐ZIF@NA^CM^ compared to other group. Interestingly, the SF‐ZIF8^CM^ group exhibited significantly elevated expression levels of OSX, OPN, and OCN compared to the Ctrl^CM^ and SF^CM^ groups, highlighting the pivotal role of Zn^2^⁺ in facilitating osteogenic differentiation. Western blot analysis (Figure S13, Supporting Information) demonstrated increased protein levels of key osteogenic markers, including OCN, OSX, ALP, and Col‐1, in the SF‐ZIF@NA^CM^ group compared to the other groups. These results corroborate the transcriptional data, further confirming the strong osteoinductive potential of SF‐ZIF@NA^CM^. In conclusion, both SF‐ZIF8^CM^ and SF‐ZIF@NA^CM^ demonstrate potential for enhancing BMSC‐mediated bone regeneration. While SF‐ZIF8 improves bone formation through the release of Zn^2+^, its effects are comparatively limited due to the absence of additional growth factor.^[^ ^28^ ^]^ However, SF‐ZIF@NA offers significant improvements through the incorporation of sialidase, which inhibits the formation of mOCs, maintains a substantial population of pOCs that secrete osteogenesis‐promoting growth factors, and synergizes with the release of Zn^2+^ to create a more favorable environment for bone formation. This makes SF‐ZIF@NA a more promising candidate material for the treatment of osteoporotic bone defects.

### 2.5 Effects of SF‐ZIF@NA Hydrogel on Angiogenesis Through Preserving pOCs

**Figure 5:**
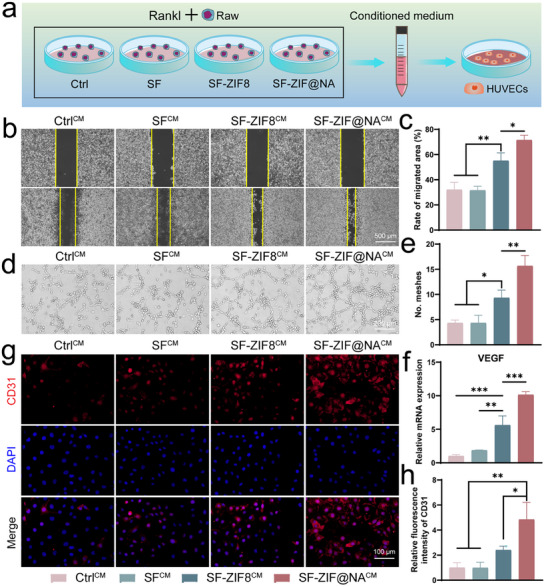
SF‐ZIF@NA preserved POC induce angiogenesis. a) Diagram exploring the effects of conditioned medium collected from RANKL‐induced macrophages treated with different hydrogels on HUVECs angiogenesis; b) Scratch assay and c) quantitative analysis using HUVECs after 24 h (n = 3, one‐way ANOVA); d) Tube formation of induced HUVECs after 6 h; e) Quantitative analysis of the number of meshes (n = 3, one‐way ANOVA); f) The relative mRNA expression levels of Angiogenesis‐related markers VEGF after 48 h of culture (n = 3, one‐way ANOVA); g) The nuclear/CD31 staining of induced HUVECs after 48 h; h) Quantitative analysis of the relative fluorescence intensity of CD31 (n = 3, one‐way ANOVA). Data are presented as mean ± SD. *p < 0.05, **p < 0.01, ***p < 0.001 indicate statistical significance.

Angiogenesis plays a crucial role in bone healing.^[^ ^29^ ^]^ To evaluate the impact of SF‐ZIF@NA‐treated osteoclasts on angiogenesis, human umbilical vein endothelial cells (HUVECs) were cultured with conditioned medium from RANKL‐induced RAW264.7 treated with different hydrogels (**Figure** **5**a). The scratch assay (Figure 5b) showed that HUVECs treated with conditioned medium from the SF‐ZIF@NA^CM^ group exhibited markedly enhanced migration after 24 h compared to other groups. Quantitative analysis (Figure 5c) revealed that the cell migration area in the SF‐ZIF@NA^CM^ group was significantly larger, ≈2.3‐fold higher than the Ctrl^CM^ group. Although the SF‐ZIF8^CM^ group also promoted cell migration compared to the Ctrl^CM^, the increase was more modest, at 1.7‐fold. These results suggest that SF‐ZIF@NA^CM^ is more effective in promoting endothelial cell migration, a critical early step in angiogenesis.^[^ ^30^ ^]^ The tube formation assay (Figure 5d) further demonstrated SF‐ZIF@NA^CM^’s potent pro‐angiogenic effects. After 6 h of incubation, HUVECs treated with SF‐ZIF@NA^CM^ formed extensive tube networks with numerous interconnected branches and closed meshes. Quantitative analysis (Figure 5e; Figure S14, Supporting Information) confirmed that both SF‐ZIF8^CM^ and SF‐ZIF@NA^CM^ significantly increased the number of meshes and master junctions compared to the Ctrl^CM^ group. To further confirm SF‐ZIF@NA^CM^’s positive influence on angiogenesis, real‐time quantitative PCR (qRT‐PCR) was used to assess the mRNA expression of VEGF, a key angiogenic marker (Figure 5f). The SF‐ZIF8^CM^ group demonstrated elevated VEGF levels compared to the Ctrl^CM^, suggesting that the release of Zn^2+^ from ZIF‐8 plays a significant role in upregulating angiogenic factors. VEGF expression in the SF‐ZIF@NA^CM^ group was the highest among all groups, with the presence of pOCs in SF‐ZIF@NA^CM^ further enhancing the angiogenic effect by secreting pro‐angiogenic factors such as PDGF‐BB. Immunofluorescence staining for CD31, a well‐known endothelial cell marker associated with angiogenesis, confirmed enhanced angiogenesis in the SF‐ZIF@NA^CM^ group. As shown in Figure 5g, HUVECs treated with SF‐ZIF@NA^CM^ exhibited stronger CD31 staining after 48 h. Quantitative analysis of fluorescence intensity (Figure 5h) demonstrated that CD31 expression was notably higher in the SF‐ZIF@NA^CM^ group compared to the other groups, further supporting the superior pro‐angiogenic potential of SF‐ZIF@NA^CM^. The enhanced angiogenesis observed with the SF‐ZIF@NA^CM^ can be attributed to several factors. First, Zn^2+^ released from ZIF‐8 are known to stimulate endothelial cell proliferation, migration, and tube formation.^[^ ^31^ ^]^ Zn^2+^ as a cofactor for various enzymes involved in angiogenesis and directly influences the expression of key genes like VEGF, as evidenced by our findings. Additionally, SF‐ZIF@NA inhibits the formation of mature osteoclasts, preserves pOCs, which secrete PDGF‐BB, a potent pro‐angiogenic factor. The combination of Zn^2+^ release and the maintenance of pro‐regenerative pOCs creates a favorable microenvironment for endothelial cell function, promoting angiogenesis. This is particularly important in tissue engineering, where achieving a balance between bone resorption and vascularization is critical for successful tissue integration and regeneration.

### 2.6 pOCs Preserved by SF‐ZIF@NA Hydrogel Induce Osteogenesis and Angiogenesis via FAK Pathway

**Figure 6:**
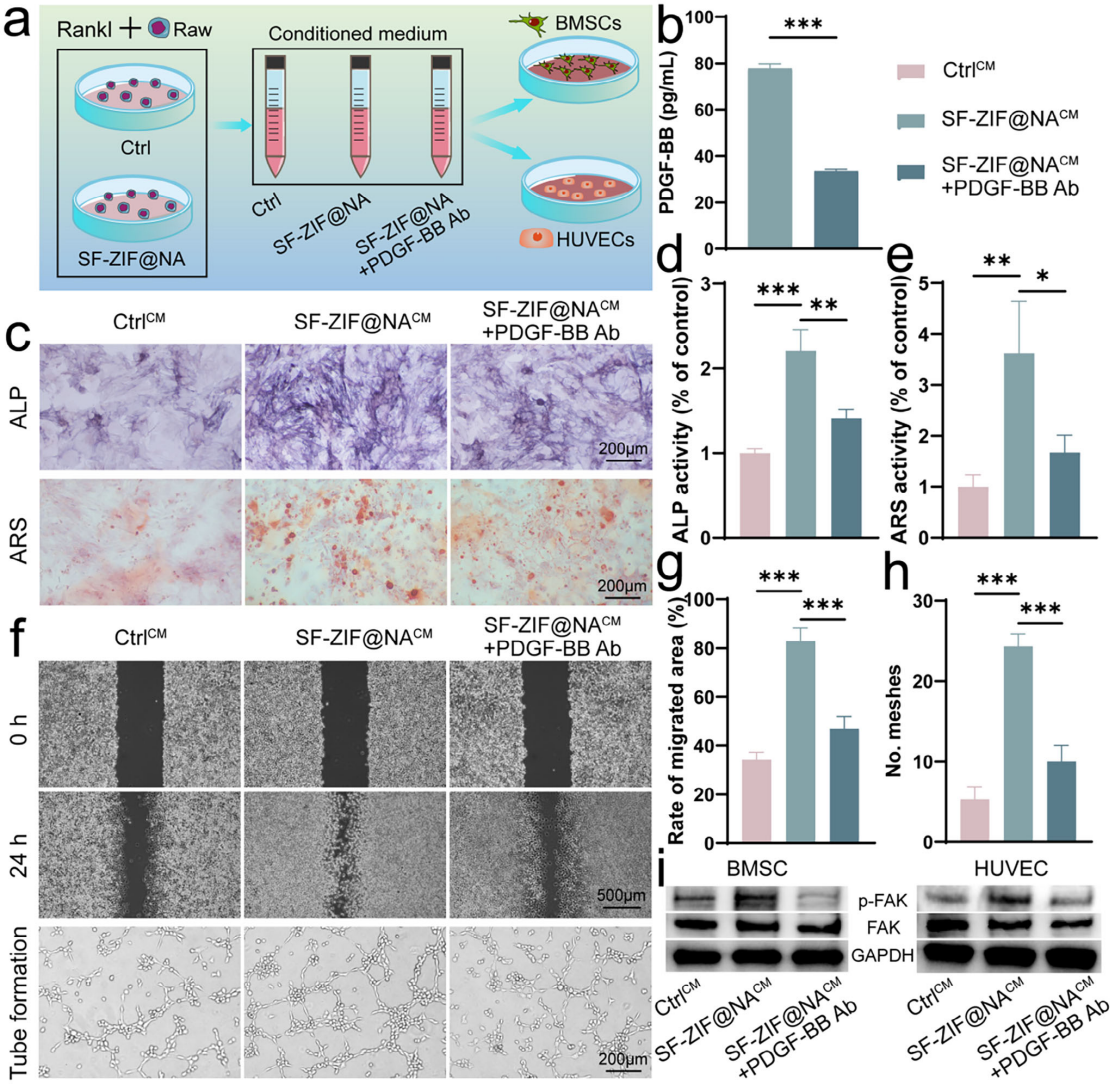
Impact of PDGF‐BB neutralizing antibody addition on osteogenic differentiation and angiogenesis. a) Diagram depicting the impact of conditioned medium with or without PDGF‐BB neutralizing antibody treatment on BMSCs osteogenic differentiation and HUVECs angiogenesis; b) Detection of PDGF‐BB in conditioned medium with or without PDGF‐BB neutralizing antibody treatment using ELISA (n = 3, unpaired Student's t‐test); c) ALP and ARS staining of BMSCs under different treatments; Quantitative analysis of d) ALP and e) ARS in BMSCs under different treatments (n = 3, one‐way ANOVA); f) Scratch assay and tube formation assay of HUVECs under different treatments; Quantitative analysis of g) migration rate and h) number of meshes (n = 3, one‐way ANOVA); i) Western blot analysis of P‐FAK levels in BMSCs and HUVECs under different culture conditions. Data are presented as mean ± SD. *p < 0.05, **p < 0.01, ***p < 0.001 indicate statistical significance.

To explore the pivotal role of PDGF‐BB secreted by pre‐osteoclasts in osteogenic differentiation and angiogenesis, we examined the effects of adding a PDGF‐BB neutralizing antibody to the conditioned medium (**Figure** **6**a). The presence of PDGF‐BB in the conditioned medium was first confirmed using ELISA (Figure 6b). As expected, the medium from SF‐ZIF@NA‐treated contained high levels of PDGF‐BB, which were significantly reduced upon treatment with the PDGF‐BB neutralizing antibody. This confirmed the effective depletion of PDGF‐BB.

The osteogenic potential of BMSCs was assessed via ALP and ARS staining, which are markers of early and late osteogenic differentiation, respectively.^[^ ^32^ ^]^ As shown in Figure 6c, BMSCs cultured in the SF‐ZIF@NA^CM^ group exhibited the highest levels of both ALP and mineralization. Quantitative analysis (Figure 6d,e) demonstrated that ALP activity was significantly higher in the SF‐ZIF@NA^CM^ group (2.2‐fold over the Ctrl^CM^), while the ARS staining showed a similar trend with a 3.6‐fold increase, suggesting enhanced osteogenic differentiation. However, in the presence of the PDGF‐BB neutralizing antibody, both ALP and ARS levels were substantially reduced. These findings suggest that PDGF‐BB, secreted by pOCs, significantly enhances the osteogenic differentiation of BMSCs, and the reduction in differentiation observed after PDGF‐BB neutralization underscores the importance of this growth factor in osteogenesis.^[^ ^33^ ^]^

To assess the effects on angiogenesis, we performed a scratch assay and tube formation assay on HUVECs (Figure 6f). In the scratch assay, HUVECs treated with the SF‐ZIF@NA^CM^ exhibited significantly enhanced migration compared to controls, with a 2.4‐fold increase in migration rate (Figure 6g). However, after neutralizing PDGF‐BB, this migration was markedly reduced, suggesting that PDGF‐BB plays an essential role in endothelial cell migration. In the tube formation assay, HUVECs treated with the SF‐ZIF@NA^CM^ formed more complex and interconnected capillary‐like structures, as evidenced by a higher number of meshes and master junctions (Figure 6h; Figure S15, Supporting Information). Again, neutralization of PDGF‐BB led to a significant reduction in tube formation. These findings highlight the essential role of PDGF‐BB in promoting endothelial cell angiogenesis, further supporting its key contribution to the pro‐angiogenic microenvironment induced by SF‐ZIF@NA‐treated osteoclasts.

To further elucidate the underlying mechanisms, we analyzed the phosphorylation of focal adhesion kinase (FAK), a well‐established critical regulator of both osteogenic differentiation and angiogenesis.^[^ ^34^ ^]^ Western blot analysis (Figure 6i) revealed that both BMSCs and HUVECs cultured in the SF‐ZIF@NA^CM^ exhibited significantly elevated P‐FAK levels compared to Ctrl^CM^, indicating strong activation of FAK signaling. However, PDGF‐BB neutralization reduced P‐FAK levels in both cell types, suggesting that PDGF‐BB secreted by pOCs preserved through SF‐ZIF@NA treatment promote osteogenic differentiation and angiogenesis by activating the FAK signaling pathway.

### 2.7 SF‐ZIF@NA Hydrogel Promotes the Repair of Osteoporotic Bone Defects

**Figure 7:**
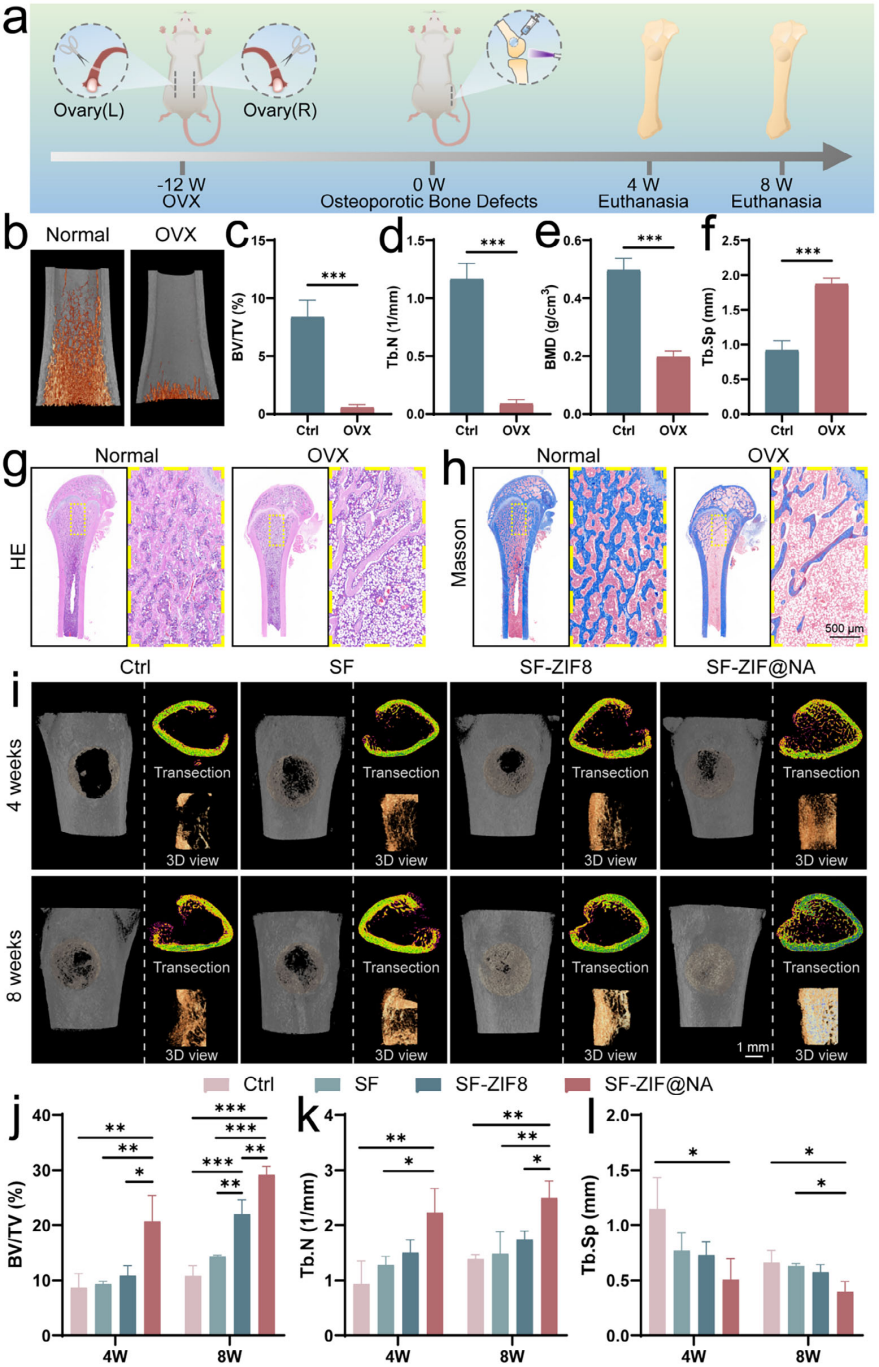
In vivo osteopromoting properties of the hydrogel in osteoporotic rats. a) Schematic diagram of the construction of an osteoporotic bone defect model in SD rats and its treatments; b) Micro‐CT images of femurs from normal and OVX; Quantitative analysis of c) BV/TV, d) Tb.N, e) BMD, and f) Tb.Sp in normal and OVX rats (n = 3, unpaired Student's t‐test); g) H&E staining and h) Masson staining of femurs from normal and OVX rats; i) 3D micro‐CT images of femoral defects at 4 and 8 weeks post‐implantation; Quantitative analysis of femoral defects after 4 and 8 weeks of different treatments: j) BV/TV, k) Tb.N, and l) Tb.Sp (n = 3, one‐way ANOVA). Data are presented as mean ± SD. *p < 0.05, **p < 0.01, ***p < 0.001 indicate statistical significance.

Osteoporotic bone defects present a significant challenge for healing due to the complex microenvironment, which necessitates specialized therapeutic interventions.^[^ ^35^ ^]^ To evaluate the bone regeneration potential of the SF‐ZIF@NA hydrogel under osteoporotic conditions, we conducted a series of in vivo studies using an osteoporotic bone defect model in SD rats. As illustrated in **Figure** **7**a, an osteoporotic model was established through bilateral ovariectomy (OVX), which induces estrogen deficiency and mimics postmenopausal osteoporosis. 12 weeks post‐surgery, micro‐CT scans were performed to confirm the successful establishment of osteoporosis. Significant osteoporotic changes were observed in the OVX group, including a marked reduction in bone volume compared to Normal group (Figure 7b). Quantitative analysis of key bone parameters (Figure 7c‐f), including bone volume fraction (BV/TV), trabecular number (Tb.N), and bone mineral density (BMD), revealed substantial decreases in the OVX group relative to the Normal group. Additionally, trabecular separation (Tb.Sp), an inverse indicator of bone health, increased significantly in OVX rats, further confirming the osteoporotic condition. These micro‐CT findings were corroborated by histological analyses using H&E and Masson staining (Figure 7g,h), which confirmed the reduction in trabecular bone in the OVX group. To further confirm the osteoporotic model, TRAP staining was performed (Figure S16, Supporting Information), revealing increased osteoclast activity in the OVX group, consistent with the pathological bone resorption characteristic of osteoporosis. Given the central role of osteoclasts in osteoporosis progression, we further investigated osteoclast differentiation in BMMs from both normal and OVX rats. TRAP (Figure S17, Supporting Information) and nuclear/F‐actin (Figure S18, Supporting Information) staining revealed no significant differences between the two groups, likely due to the absence of estrogen in the in vitro culture environment, which eliminated the hormonal influence that typically amplifies osteoclastogenesis in OVX models. Importantly, SF‐ZIF@NA effectively inhibited osteoclast differentiation in BMMs, consistent with its inhibitory effects observed in RAW264.7 cells. These findings underscore the therapeutic consistency and broad applicability of SF‐ZIF@NA in suppressing osteoclast activity, demonstrating its potential as a therapeutic strategy for osteoporosis treatment.

Following the verification of osteoporosis, an osteoporotic bone defect model was created by introducing cylindrical defects (3 mm diameter, 2 mm depth) in the distal femurs of OVX rats (Figure 7a; Figure S19, Supporting Information). The bone defects were treated with different hydrogels (Ctrl, SF, SF‐ZIF8, and SF‐ZIF@NA), and in situ crosslinking was achieved using UV light. Bone regeneration within the osteoporotic defect regions was evaluated at 4 and 8 weeks post‐treatment using micro‐CT (Figure 7i). 3D reconstructions of the CT images revealed new bone formation in all groups. At 4 weeks, both the SF‐ZIF8 and SF‐ZIF@NA groups exhibited smaller defect areas compared to the Ctrl and SF groups. By 8 weeks, all groups demonstrated more extensive bone formation, with the SF‐ZIF@NA group showing near‐complete defect coverage by new bone. The 3D view corroborated this trend. Notably, cross‐sectional CT images revealed that at both 4 and 8 weeks, the SF‐ZIF@NA group exhibited not only greater cortical bone formation but also a more substantial amount of trabecular bone compared to the other groups. This effect can likely be attributed to the NA component, which inhibits osteoclast fusion, thereby mitigating the progression of osteoporosis. Quantitative analysis of key bone parameters, including BV/TV, Tb.N, and Tb.Sp, showed that the SF‐ZIF@NA group had significantly higher BV/TV and Tb.N values, along with lower Tb.Sp values, compared to the other groups (Figure 7j‐l). In conclusion, these findings highlight the remarkable osteoinductive properties of the SF‐ZIF@NA hydrogel in the treatment of osteoporotic bone defects. While the SF‐ZIF8 hydrogel facilitates bone regeneration through the release of Zn^2+^, the incorporation of NA in the SF‐ZIF@NA hydrogel significantly enhances its therapeutic efficacy by suppressing excessive osteoclast activity and preserving the bone microenvironment.

### 2.8 Histological Evaluation of SF‐ZIF@NA Hydrogel for Treating Osteoporotic Bone Defects

**Figure 8:**
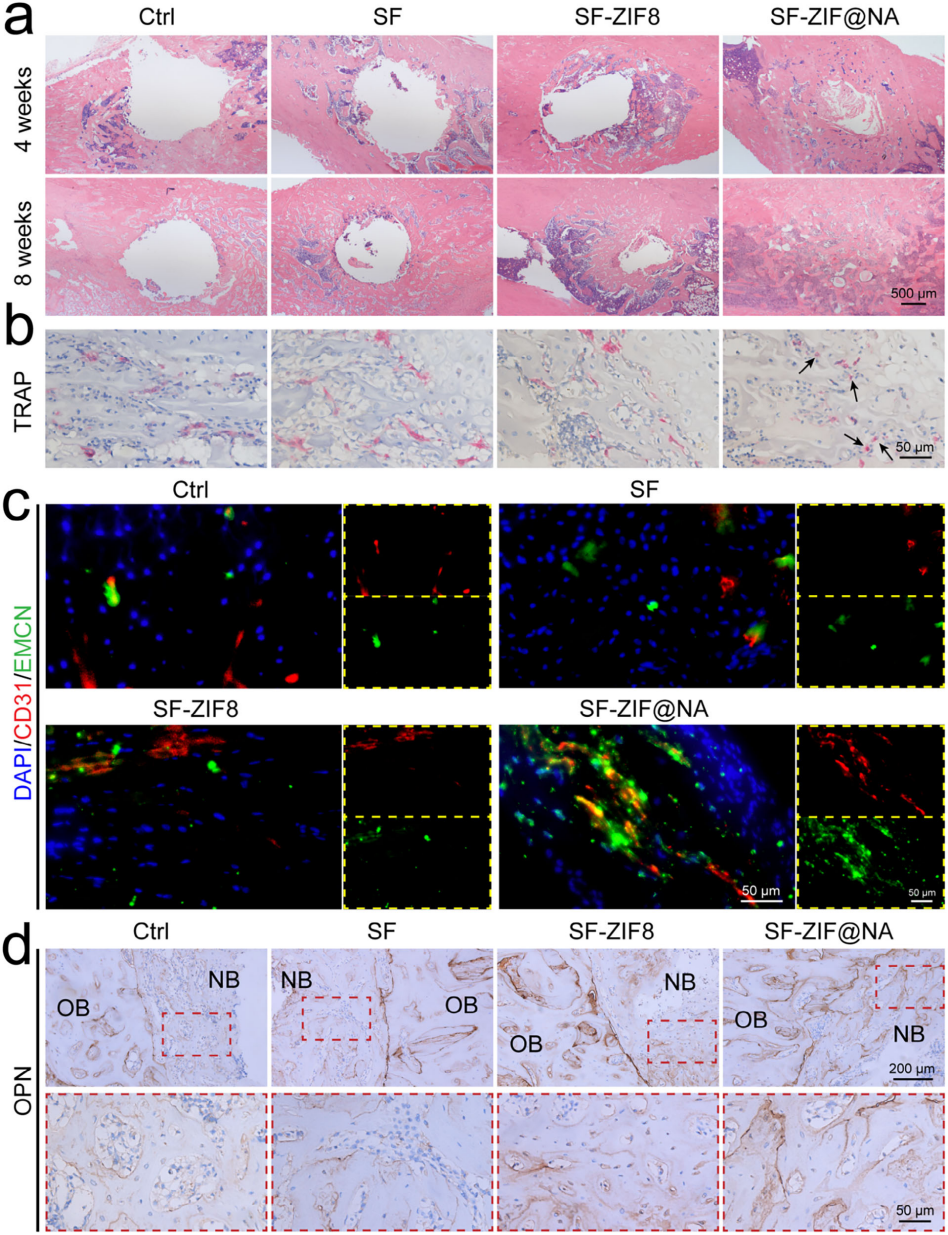
Histopathological and immunohistochemical data of osteoporotic bone defects. a) HE staining of femoral defects at 4 and 8 weeks post‐implantation; b) TRAP staining of femoral defects at 4 weeks post‐implantation; c) The nuclear/CD31/EMCN staining of femoral defects 8 weeks post‐implantation; d) Immunohistochemical staining of the osteogenic marker OPN in femoral defects 8 weeks post‐implantation, OB denotes the original bone tissue, and NB signifies the newly formed bone tissue.

To further evaluate the newly formed bone at 4 and 8 weeks post‐implantation, a series of histological analyses were conducted. Hematoxylin and eosin (H&E) staining was used to assess the morphological features of bone defects and the extent of bone regeneration. As shown in **Figure** **8**a, at 4 weeks post‐operation, the defect in the Ctrl group remained pronounced, while the SF‐ZIF@NA group exhibited the most significant new bone formation. SF‐ZIF@NA group demonstrated the fastest and most effective healing compared to other groups. By 8 weeks post‐implantation, a mature and stable bone structure was observed in the SF‐ZIF@NA group, with complete integration between the host bone and newly formed bone. The absence of a distinct boundary between the two further confirms that SF‐ZIF@NA significantly enhanced bone regeneration, consistent with the micro‐CT results. TRAP staining was used to assess osteoclast activity (Figure 8b; Figure S20, Supporting Information). The SF‐ZIF@NA group showed a notable reduction in the number of multinucleated osteoclasts on the trabecular surface compared to other groups, while maintaining more pOCs (indicated by black arrows). This suggests that SF‐ZIF@NA may inhibit excessive osteoclast‐mediated bone resorption, promoting a more favorable environment for bone regeneration.^[^ ^36^ ^]^ Recent studies have identified a distinct subtype of blood vessels, known as H‐type vessels (CD31^hi^Emcn^hi^), which are closely associated with new bone formation.^[^ ^37^ ^]^ The development of these vessels is induced by PDGF‐BB, secreted by pOCs.^[^ ^26^, ^38^ ^]^ Immunofluorescence staining for PDGF‐BB (Figure S21, Supporting Information) revealed a significantly higher expression of PDGF‐BB in the SF‐ZIF@NA group compared to the other groups. The formation of H‐type vessels was further investigated through immunofluorescent staining for Emcn and CD31. As shown in Figure 8c and Figure S22 (Supporting Information), the SF‐ZIF@NA group exhibited a marked increase in CD31^hi^Emcn^hi^ double‐positive vessels, signifying enhanced H‐type vessel formation compared to other groups. Although the SF‐ZIF8 group did not show a notable increase in Emcn expression, it did present more CD31‐positive vessels than the blank and SF groups. This is likely attributable to the angiogenic effects of Zn^2⁺^, which, while promoting general angiogenesis, do not specifically enhance the formation of H‐type vessels.^[^ ^34^ ^]^ Angiogenesis is critical for bone formation, and the generation of H‐type vessels is especially important in osteoporotic environments, where insufficient vascularization often limits bone repair.^[^ ^39^ ^]^ OPN, a critical marker of osteogenesis, was assessed through immunohistochemical staining (Figure 8d; Figure S23, Supporting Information). The SF‐ZIF@NA group exhibited the highest levels of OPN expression at 8 weeks post‐implantation, with levels approaching those observed in normal bone tissue. These findings further support the conclusion that the SF‐ZIF@NA hydrogel system effectively promotes bone regeneration under osteoporotic conditions. Furthermore, HE staining of major organs was used to evaluate tissue toxicity (Figure S24, Supporting Information), and no significant differences were observed among all groups, indicating that the hydrogel did not induce noticeable systemic toxicity in vivo and possesses good biocompatibility. In summary, even in the challenging pathological context of osteoporosis, the SF‐ZIF@NA hydrogel system demonstrated the capacity to modulate the local tissue microenvironment, promoting both osteogenesis and angiogenesis, particularly the formation of H‐type vessels. These combined effects resulted in a significantly accelerated bone regeneration rate, highlighting the potential of this system for treating osteoporotic bone defects.

## 3 Conclusion

This study introduces an innovative SF‐ZIF@NA hydrogel, which employs a trifunctional strategy to address the complex challenges of osteoporotic bone healing. By precisely modulating osteoclast activity, SF‐ZIF@NA inhibits excessive bone resorption while preserving the anabolic function of pOCs, thereby enhancing coupled osteogenesis and angiogenesis. Specifically, SF‐ZIF@NA prevents pOC‐to‐mOC fusion via sialic acid removal, maintaining pOC‐secreted PDGF‐BB to support vascularized bone regeneration. Additionally, the hydrogel releases Zn^2^⁺ in response to acidic conditions, further stimulating osteogenic differentiation and improving the bone‐healing microenvironment. In vivo, SF‐ZIF@NA hydrogel significantly accelerated bone defect repair in osteoporotic models, enhancing bone density, structural integrity, and vascularization without systemic toxicity. These findings suggest that a synergistic strategy combining anabolic preservation with ion‐mediated effects offers a promising, targeted solution to the complex challenges of osteoporotic bone regeneration.

## 4 Experimental Section

### Preparation of ZIF‐8 and ZIF@NA

To prepare ZIF@NA, 970 mg of 2‐Methylimidazole (Aladdin, China) was dispersed in 9 mL of ultrapure water, followed by the addition of 1 mL Neuramindase (NA, Aladdin, China) solution (5 mg mL^−1^), with gentle stirring for 15 min. Next, 10 mL of Zinc acetate solution (3.8 mg mL^−1^) was introduced and stirred for 12 h. The resulting product, ZIF@NA, was collected via centrifugation and washed thoroughly. Pure ZIF‐8 was synthesized under the same conditions without NA inclusion.

### Preparation of SF‐ZIF and SF‐ZIF@NA

Following modifications of previous protocols, silk fibroin methacryloyl (SFMA) was synthesized.^[^ ^40^ ^]^ Briefly, silkworm cocoons were cut into pieces and boiled in a 2 g L^−1^ sodium carbonate solution for 1 h to remove sericin. The degummed silk fibroin (2 g) was dissolved in 10 mL of 9.3 M lithium bromide solution, heated at 60 °C for 6 h, then slowly added to 350 mM glycidyl methacrylate (GMA, Aladdin, China) with stirring at 300 rpm for 3 h. The solution was transferred to a dialysis bag, dialyzed in distilled water for five days, and lyophilized to obtain SFMA. For hydrogel preparation, a 15% SFMA solution with LAP photoinitiator (0.1% w/v) was heated to 60 °C, followed by the addition of either ZIF‐8 or ZIF@NA (refer to Table S2, Supporting Information for ratios) and mixed. The hydrogel was formed by UV irradiation (405 nm, 5 W) for 30 s. SFMA and the photoinitiator alone constituted the SF hydrogel.

### Characterization of ZIF@NA and SF‐ZIF@NA

ZIF@NA samples were deposited onto carbon‐copper grids and observed using a transmission electron microscope (TEM, FEI‐TecnaiG2F20, USA) equipped with X‐ray spectroscopy. Crystallographic analysis was conducted via X‐ray diffraction (XRD, MiniFlex600, Japan). The Specific surface area and pore size analyzer (BET, ASAP2460) method was used for surface area and pore analysis. Particle size distribution was analyzed by dynamic light scattering (DLS), and zeta potential was measured in water dispersion. Cy5.5‐labeled neuraminidase in ZIF@NA allowed fluorescence imaging of enzyme distribution via confocal microscopy (ZISS 880, Germany). To assess enzyme activity under varying pH conditions, free neuraminidase and ZIF@NA were prepared in PBS at different pH levels, maintained for 24 h, and enzyme activity was measured with a neuraminidase activity kit (Aladdin, China). Stability tests in water were conducted by measuring enzyme activity over time. Methacrylation levels of SFMA were determined using 1H‐NMR, while freeze‐dried hydrogel morphology and elemental distributions were observed via scanning electron microscopy (SEM, SU‐8020, Japan) and energy dispersive X‐ray spectroscopy (EDX). The mechanical properties of the hydrogel (5 mm in diameter and 3.5 mm in height) were evaluated using a universal testing machine (Instron 5943, Instron, USA), with the hydrogel being compressed to 80%. To determine the degradation rate of SF‐ZIF@NA hydrogel, lyophilized samples were immersed in culture media with different pH values (pH 7.4 and pH 5.5) to reach swelling equilibrium. The initial mass was measured and recorded as W0. At predetermined time intervals, excess water was removed from the samples using filter paper, and the mass was measured and recorded as Wt. The degradation rate was calculated using the following formula: Degradation ratio (%) = [(W_0_‐W_t_)/W_0_]*100%.

### Cell Culture

Bone marrow mesenchymal stem cells (BMSCs) were extracted from Sprague‐Dawley (SD) rats (3–5 days old) following the ethical guidelines of Army Medical University.^[^ ^41^ ^]^ Cells were cultured in α‐MEM medium (Gibco, USA) with 10% fetal bovine serum (FBS, Zhejiang Tianhang Biotechnology Ltd., China) and 1% penicillin‐streptomycin (Solarbio, China), with media changes every three days. At 80–90% confluence, cells were passaged with trypsin, and BMSCs from passages 2–5 were used in experiments. RAW264.7 macrophages and HUVEC endothelial cells were cultured in DMEM (Pricella, China) supplemented with 5% FBS and 1% penicillin‐streptomycin at 37 °C and 5% CO₂.

### Biocompatibility Evaluation

Cell viability was determined using the CCK‐8 assay (Beyotime, China). Briefly, 5 × 10⁴ BMSCs were seeded in 24‐well plates and cultured for 24 h, followed by the addition of 300 µL of the photocured hydrogel for co‐culturing at 1, 3, and 7 days. After washing, cells were incubated in fresh medium containing 10% CCK‐8 at 37 °C for 1.5 h. Optical density was measured at 450 nm. Calcein‐AM/PI staining (Solarbio, China) was used to identify live/dead cells, and fluorescence microscopy images were captured, with live cells appearing green and dead cells red. Quantitative analysis was conducted using ImageJ software. To assess in vivo safety, the hydrogel was implanted in the bone defects of SD rats, with histological analysis performed on heart, liver, spleen, lungs, and kidneys after eight weeks.

### Osteoclast Differentiation In Vitro

RAW264.7 macrophages were differentiated into osteoclasts using RANKL (R&D Systems, USA) to investigate the effects of different hydrogels on osteoclastogenesis. Briefly, 5 × 10^3^ RAW264.7 cells were seeded in 24‐well plates with 1 mL DMEM complete medium and cultured for 12 h. After removing the DMEM, fresh DMEM containing RANKL (100 ng mL^−1^) and 300 µL of UV‐cured hydrogel was added. On the 3th day, cells were fixed with 4% paraformaldehyde for 15 min and stained using a sialidase staining kit. On the 7th day, cells were washed three times with PBS, fixed, and stained with Actin‐Tracker Green‐488 for cytoskeletal visualization and DAPI for nuclear staining. Cells underwent TRAP staining using a tartrate‐resistant acid phosphatase (TRAP) activity staining kit (Jemmy Gene, China) at 37 °C for 60 min, followed by deionized water washing to terminate the reaction. Microscopy was used to capture images, with ImageJ software performing quantitative analysis. The pit formation assay was conducted by inducing RAW264.7 cells with osteoclastic differentiation medium and hydrogels for 10 days. Calf bone slices were washed with a sodium hypochlorite bleach solution. Afterward, the bone slices were stained with Toluidine Blue and observed under a microscope for imaging. Osteoclastic gene expression was evaluated using qRT‐PCR. RNA extraction utilized a RNA‐Quick Purification Kit (Yishan Biological, China), and cDNA synthesis was followed by qRT‐PCR analysis on a LightCycler 480 system (Roche, Germany). The osteoclastic genes analyzed included cathepsin K (CTSK), Fos proto‐oncogene (FOS), and matrix metallopeptidase 9 (MMP9). Additionally, PDGF‐BB levels in the culture supernatant were measured using an ELISA kit (Lianke Biological), and protein concentrations were calculated from a standard curve.

### Osteogenic Differentiation In Vitro

Collected culture media from each group were centrifuged at 2000 g for 10 min to obtain conditioned media for subsequent experiments. The osteogenic induction medium was prepared with 100 µg mL^−1^ glutamine, 10 mM β‐glycerophosphate, 50 µM ascorbate, and 10 nM dexamethasone. To evaluate osteogenic differentiation, ALP activity and mineral deposition were assessed. BMSCs (5 × 10⁴ cells) were seeded in 24‐well plates with a mixture of conditioned medium and osteogenic induction medium (1:1). After 7 days of induction, ALP activity was evaluated using a BCIP/NBT ALP kit (Beyotime Biological, China). On the 14th day, cells were fixed with 4% paraformaldehyde for 15 min and stained with Alizarin Red solution (Beyotime Biological, China) to visualize mineralized nodules. RUNX2 protein expression was detected by immunofluorescence, with cells fixed for 15 min after 7 days of induction, blocked with goat serum, and incubated with primary anti‐RUNX2 antibodies overnight at 4 °C. Secondary antibodies and FITC‐labeled probes were used for 1‐h incubation in the dark, followed by DAPI nuclear staining. Osteogenic gene expression, including collagen type I (COL1A1), osteocalcin (OCN), alkaline phosphatase (ALP), runt‐related transcription factor 2 (RUNX2), osteopontin (OPN), and Osterix (OSX), was measured by qRT‐PCR using BIO‐RAD CFX96 fluorescence PCR.

### Angiogenesis Evaluation In Vitro

To assess endothelial cell migration, a scratch assay was performed. 5 × 10^5^ HUVECs were seeded in 6‐well plates, and after confluence, a scratch was made using a pipette tip. The medium was replaced with a 1:1 mix of conditioned medium and DMEM, and after 24 h, images were taken with an optical microscope, with ImageJ software calculating the migration area. For tube formation assays, 300 µL of Matrigel (Corning, USA) was plated in a 24‐well plate, and 300 µL of a 4 × 10^5^ mL^−1^ HUVEC suspension was added. After 6 h of incubation, tube formation was imaged under a microscope. Angiogenic markers were analyzed using qRT‐PCR (VEGF) and immunofluorescence (CD31).

### Osteoporosis Model and Osteoporotic Bone Defect Model Construction

All animal experiments complied with the ethical guidelines of the Third Military Medical University Animal Research Ethics Committee (AMUWEC20242059). Osteoporosis was induced in female rats (weighing ≈150 g) via bilateral ovariectomy. After anesthetizing with 3% pentobarbital sodium, ovaries were removed. Three months post‐surgery, the femurs were collected for CT scanning and histological staining to evaluate bone loss in OVX rats. Following the OVX model, cylindrical defects (3 mm diameter, 2 mm depth) were created in the distal femurs of OVX rats. Hydrogels (50 µl) were injected into defects, UV‐cured for 30 s, and the wound was sutured. After 4 and 8 weeks, rats were euthanized, and femurs were fixed with 4% paraformaldehyde. CT scans and histological analyses were performed to assess bone regeneration. BMMs were isolated from the bone marrow cavity of both normal and ovariectomized (OVX) rats. The cells were seeded into 24‐well plates at a density of 1 × 10^4^ cells per well and subjected to various treatments. After 7 days of osteoclastogenesis induction, the cells were stained using a TRAP staining kit according to the manufacturer's instructions. Additionally, after 7 days of induction, cells were fixed, and the actin cytoskeleton was stained using Actin‐Tracker Red‐594 (Beyotime Biological, China), while nuclei were stained with DAPI. Images were captured using a microscope, and quantitative analysis was performed using ImageJ software.

### CT Scanning

Bone regeneration in the defect sites was evaluated using micro‐CT with a SkyScan 1272 scanner (Bruker, Belgium). Samples underwent three‐dimensional reconstruction and two‐dimensional imaging using SkyScan software (CTvox and DataViewer). A semi‐automated region of interest (ROI) was selected in the bone defect region, and parameters, including bone mineral density (BMD), bone volume fraction (BV/TV), trabecular number (Tb.N), trabecular thickness (Tb.Th), cortical thickness (Ct.Th), and trabecular separation (Tb.Sp), were quantitatively analyzed.

### Histopathological Evaluation

Samples were decalcified in EDTA for ≈40 days (with EDTA changes every 3 days), dehydrated, and embedded in paraffin. Sections (4 µm thick) were stained with hematoxylin and eosin (H&E), TRAP, and analyzed via immunohistochemical staining for osteopontin (OPN). Immunofluorescent staining for CD31, EMCN, and PDGF‐BB was also performed. Fluorescence microscopy was used for imaging.

### Statistical Analysis

Images were randomly obtained from the samples. Each experiment was performed with at least three independent samples (n = 3), and data were presented as mean ± standard deviation (SD). Statistical analysis was conducted using GraphPad Prism 8.0 software, with one‐way ANOVA for multiple comparisons and an unpaired Student's t‐test for two‐group comparisons. *p < 0.05, **p < 0.01, ***p < 0.001 indicate statistical significance, while ns indicates no significant difference.

## Conflict of Interest

The authors declare no conflict of interest.

## Author Contributions

Z.C. and W.Y. contributed equally to this work. Z.C. performed data curation, formal analysis, investigation, methodology, software, validation, visualization, wrote the original draft. W.Y. performed data curation, formal analysis, investigation, methodology, software, and validation. Y.T. performed conceptualization, funding acquisition, and investigation. Q.D. performed data curation, formal analysis, and validation. K.H. performed methodology and visualization. J.T. performed formal analysis and methodology. J.Z. performed formal analysis and methodology. J.C. performed methodology, validation, and visualization. Q.Y. performed formal analysis and methodology. Q.D. performed data curation, formal analysis and methodology. J.X. performed funding acquisition, investigation, project administration and resources. S.G. performed formal analysis, project administration, supervision, wrote, review and edited the final manuscript. C.D. performed conceptualization, formal analysis, validation, investigation, project administration, supervision, visualization, wrote, review and edited the final manuscript. F.L. performed conceptualization, funding acquisition, investigation, project administration, resources, supervision, visualization, wrote, review and edited the final manuscript.

## Supporting information



Supporting Information

Supplemental Movie 1

## Data Availability

The data that support the findings of this study are available from the corresponding author upon reasonable request.
