# Supplementary material for: Trifunctional Sialylation‐Based SF‐ZIF@NA Hydrogel for Selective Osteoclast Inhibition and Enhanced Bone‐Vessel Regeneration in Osteoporotic Bone Defects
Source: Adv Sci (Weinh). 2025 Mar 26;12(19):2415895. doi: 10.1002/advs.202415895 (PMC12097110; doi:10.1002/advs.202415895)
Supplement: Supplementary file 1 — Supporting Information [file ADVS-12-2415895-s002.docx]

Supporting Information

**Trifunctional Sialylation-Based SF-ZIF@NA Hydrogel for Selective Osteoclast Inhibition and Enhanced Bone-Vessel Regeneration in Osteoporotic Bone Defects**

*Zhengrong Chen ^a,^* ^#^*, Wenxin Yang ^a, b,^* ^#^*, Yong Tang ^a^, Qianqian Dong ^c, d^, Kui Huang ^a^, Jiulin Tan ^a^, Jie Zhang ^a^, Juan Cai ^a^, Qixiu Yu ^a^, Qijie Dai ^a^, Jianzhong Xu ^a^, Shuquan Guo ^e,^ *, Ce Dou ^a,^ **, Fei Luo ^a,^ ****


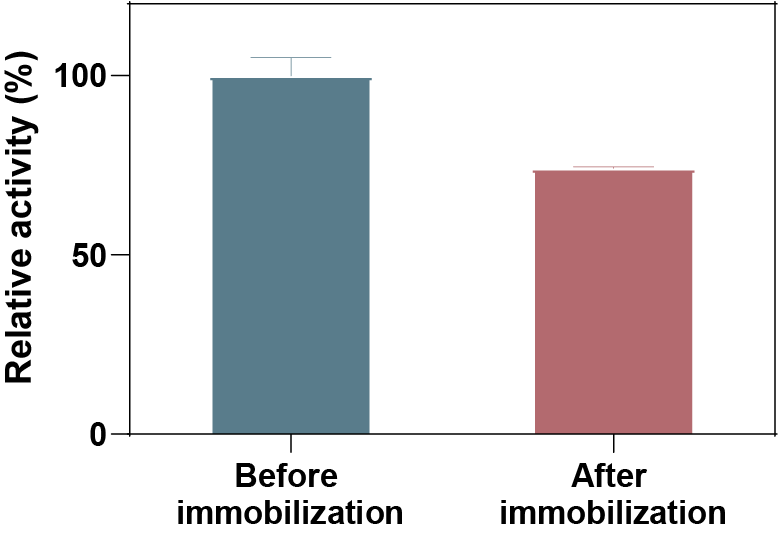


**Figure S1.** Relative enzymatic activity of supernatant before and after encapsulation. Data are presented as mean ± SD (n=3).


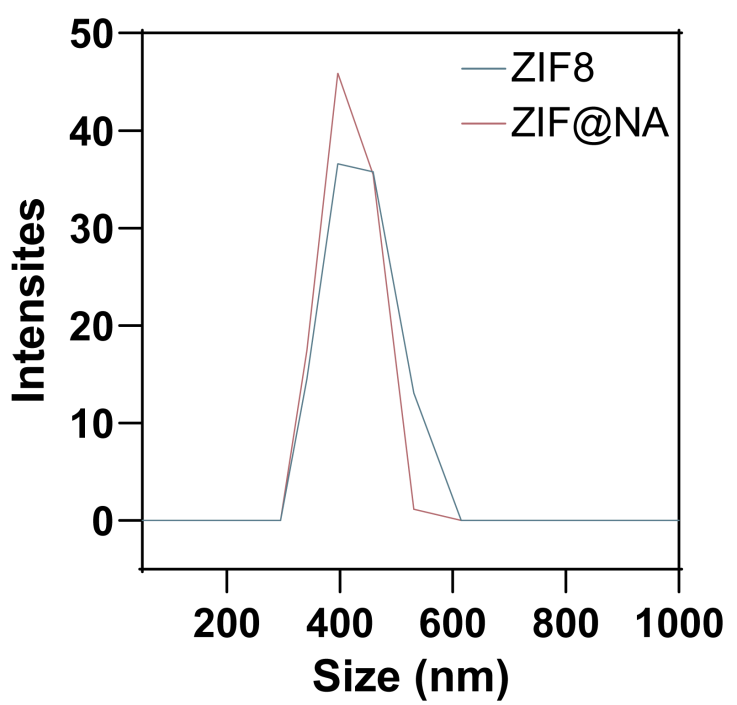


**Figure S2.** Size distribution of ZIF8 and ZIF8@NA.


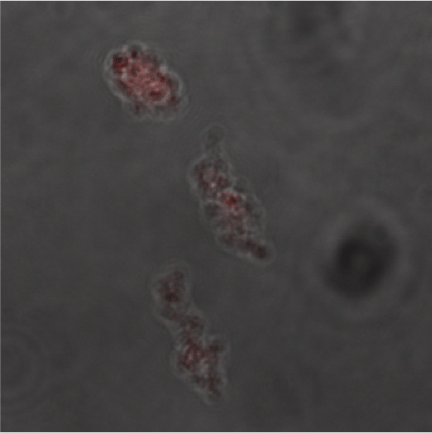


**Figure S3.** Confocal images of Cy5.5-labeled NA within ZIF@NA.


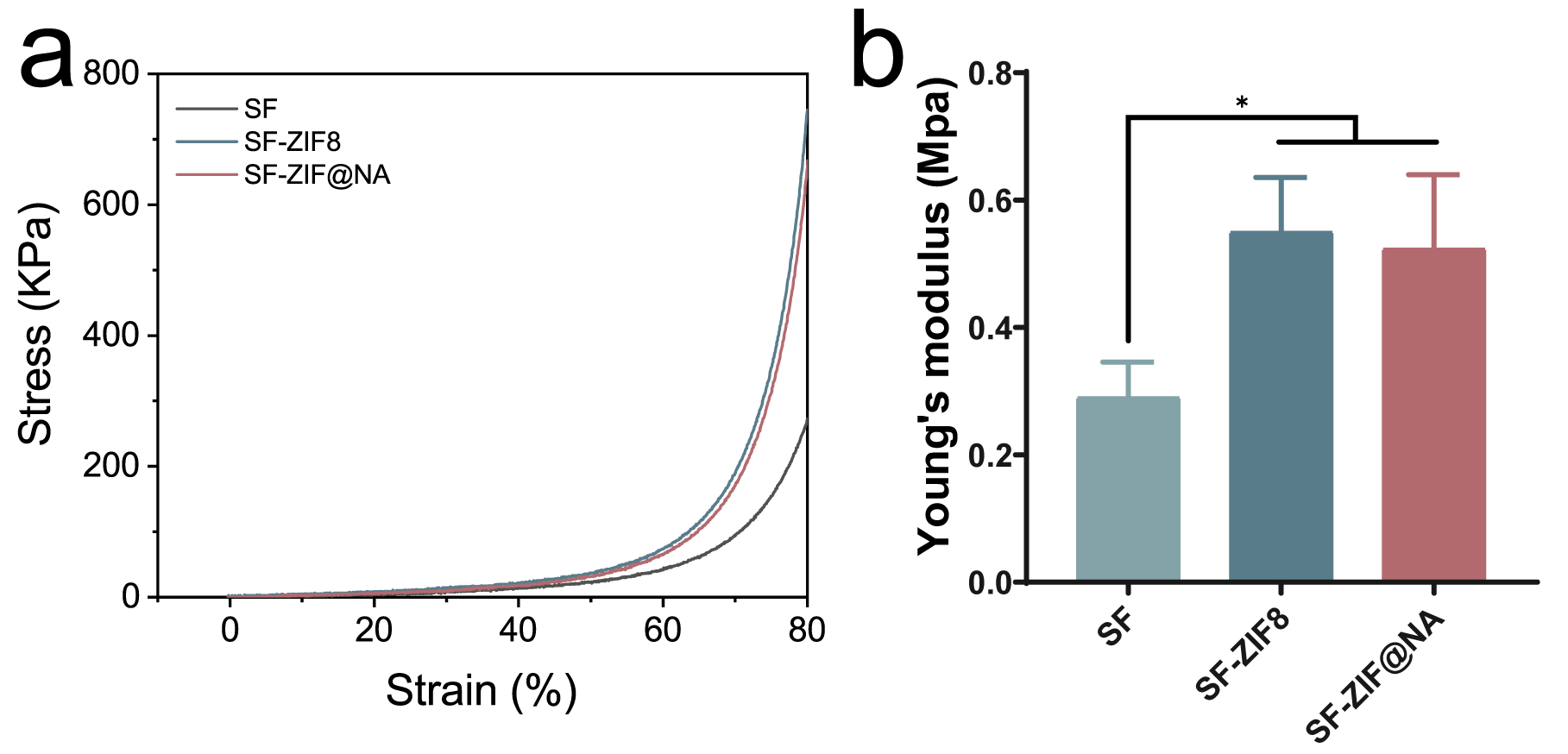


**Figure S4.** (a) Stress–strain curves during compression process and (b) Elasticity modulus of SF, SF-ZIF8, and SF-ZIF@NA. Data are presented as mean ± SD (n=3, one-way ANOVA). *p <0.05, **p <0.01, ***p <0.001 indicate statistical significance.


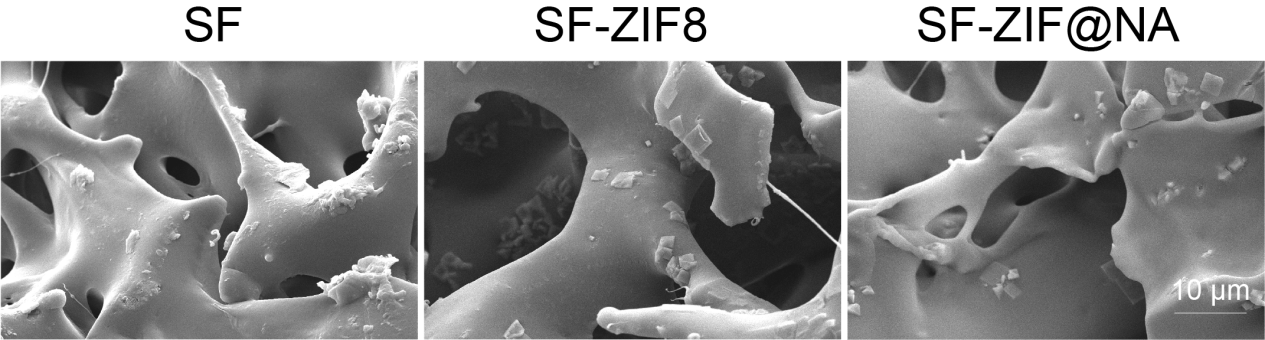


**Figure S5.** SEM images of SF, SF-ZIF8, and SF-ZIF@NA.


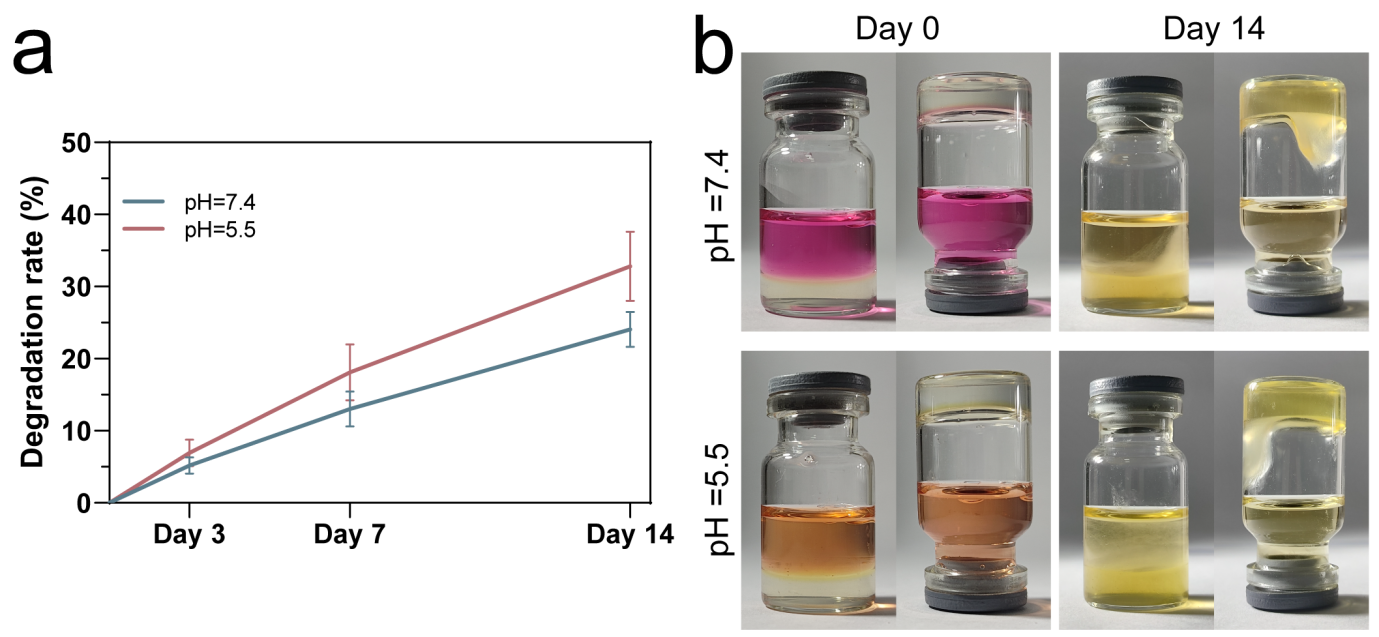


**Figure S6.** (a) Degradation Profiles of SF-ZIF@NA Hydrogel at pH 7.4 and 5.5; (b) Optical images of SF-ZIF@NA hydrogel after 14 days of degradation assay. Data are presented as mean ± SD (n=3).


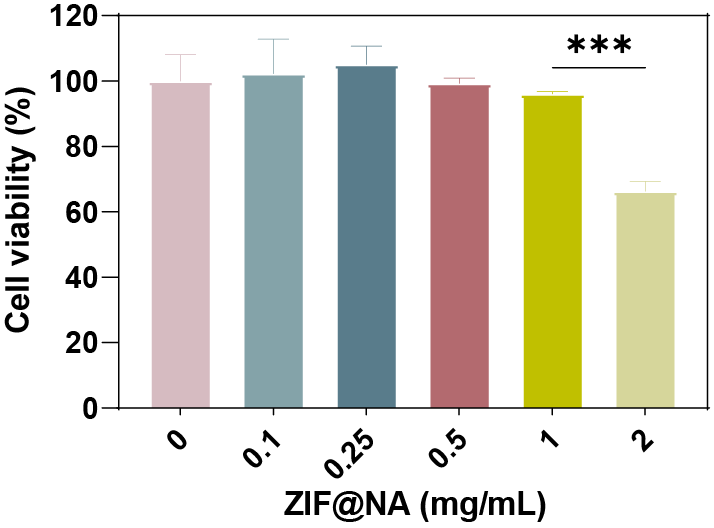


**Figure S7.** Relative viability of BMSCs in SF-ZIF@NA hydrogel with varying concentrations of ZIF@NA. Data are presented as mean ± SD (n=3, one-way ANOVA). *p <0.05, **p <0.01, ***p <0.001 indicate statistical significance.


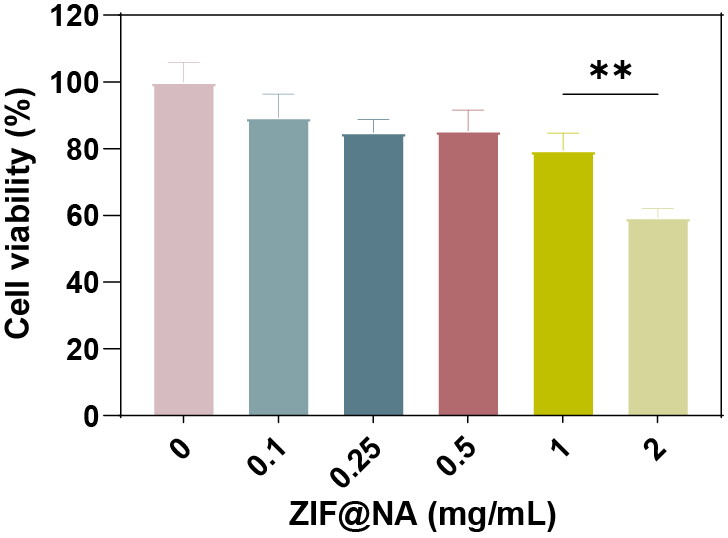


**Figure S8.** Relative viability of RAW264.7 in SF-ZIF@NA hydrogel with varying concentrations of ZIF@NA. Data are presented as mean ± SD (n=3, one-way ANOVA). *p <0.05, **p <0.01, ***p <0.001 indicate statistical significance.


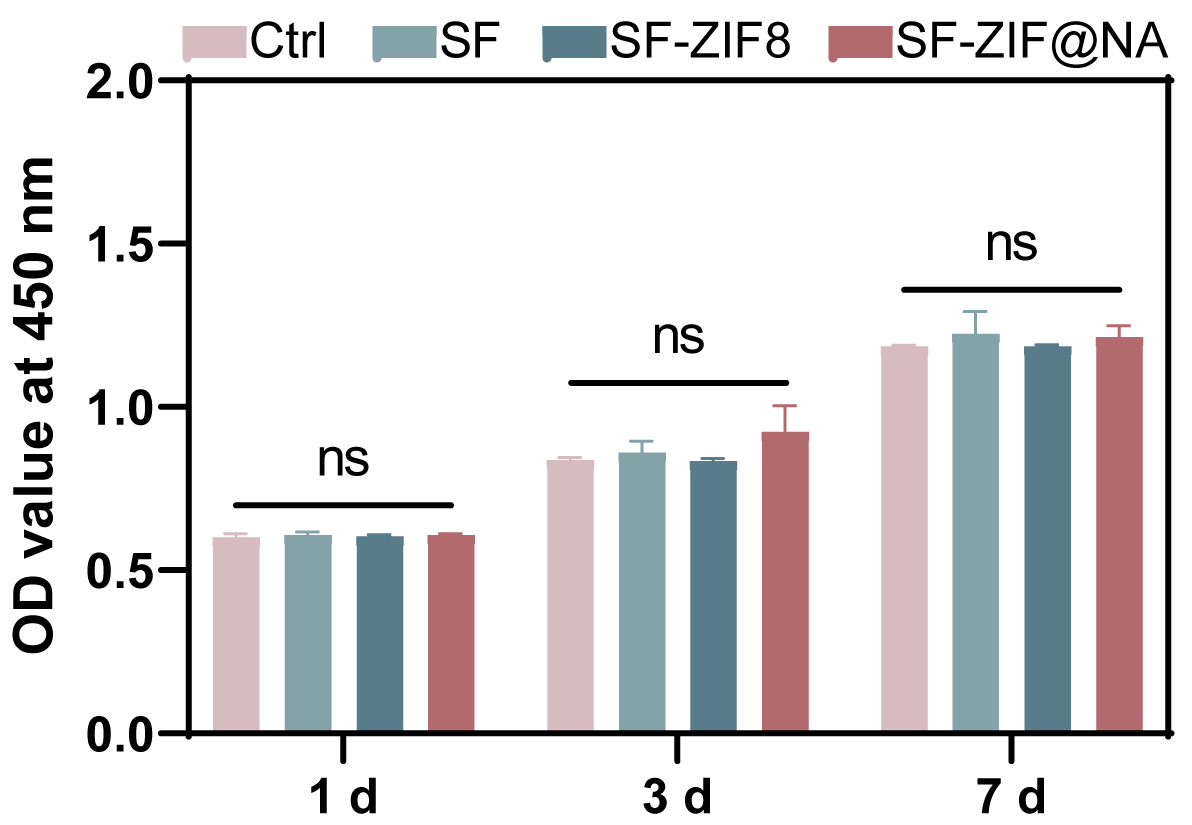


**Figure S9.** CCK-8 assay of BMSCs co-cultured with equal volumes of PBS and hydrogels after 1, 3 and 7 days. Data are presented as mean ± SD (n=3, one-way ANOVA). ns indicates no significant difference.


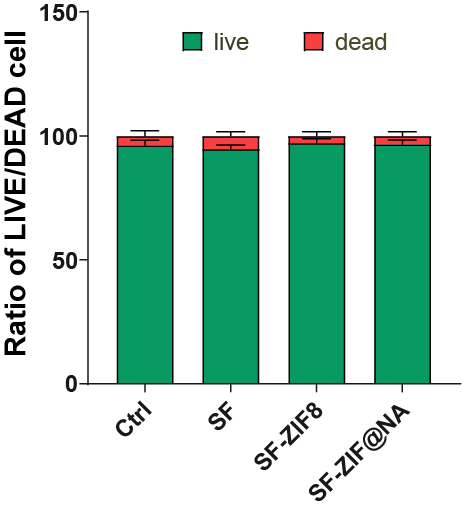


**Figure S10.** Ratio of live/dead cell in different groups. Data are presented as mean ± SD (n = 3).


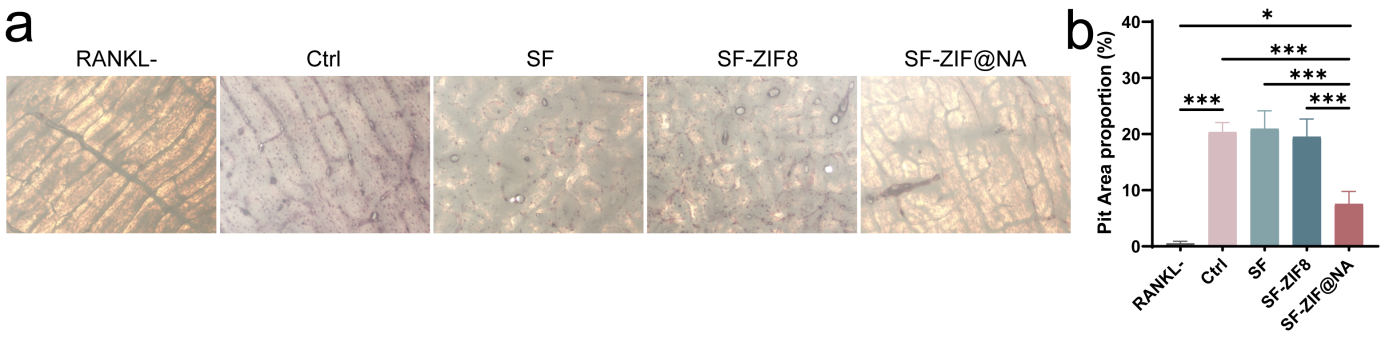


**Figure S11.** (a) Pit formation assay and (b) quantification of resorption area on bovine bone slices. Data are presented as mean ± SD (n=3, one-way ANOVA). *p <0.05, **p <0.01, ***p <0.001 indicate statistical significance.


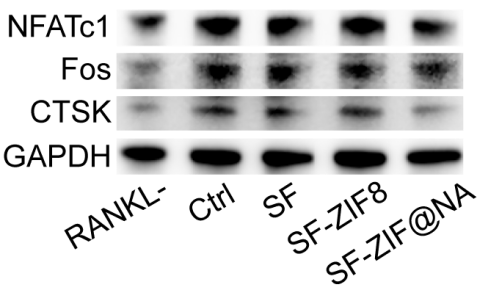


**Figure S12.** Western blot assay of protein level of CTSK, Fos, and NFATc1; GAPDH was used as a protein loading control.

**
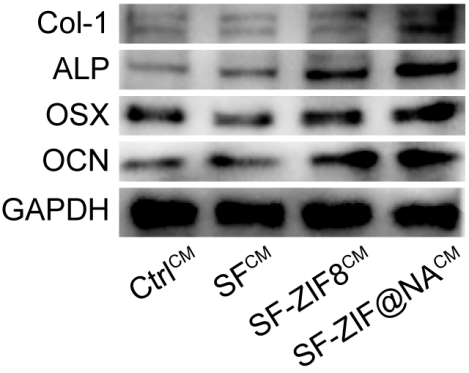
**

**Figure S13.** Western blot assay of protein level of OCN, OSX, ALP, and Col-1; GAPDH was used as a protein loading control.


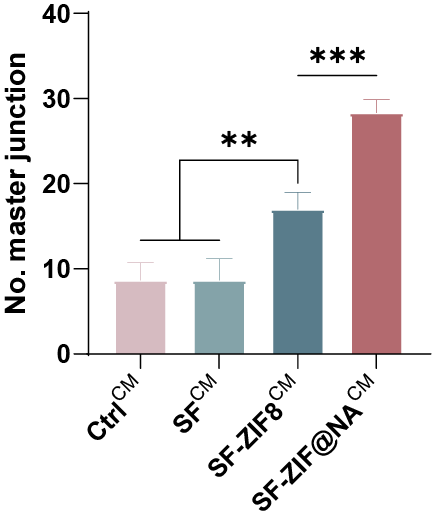


**Figure S14.** Quantitative analysis of the number of master junctions. Data are presented as mean ± SD (n=3, one-way ANOVA). *p <0.05, **p <0.01, ***p <0.001 indicate statistical significance.


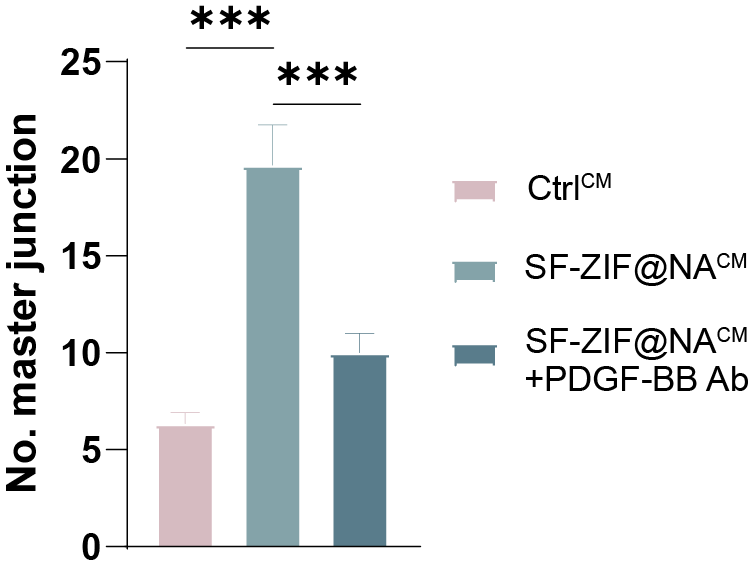


**Figure S15.** Quantitative analysis of the number of master junctions. Data are presented as mean ± SD (n=3, one-way ANOVA). *p <0.05, **p <0.01, ***p <0.001 indicate statistical significance.


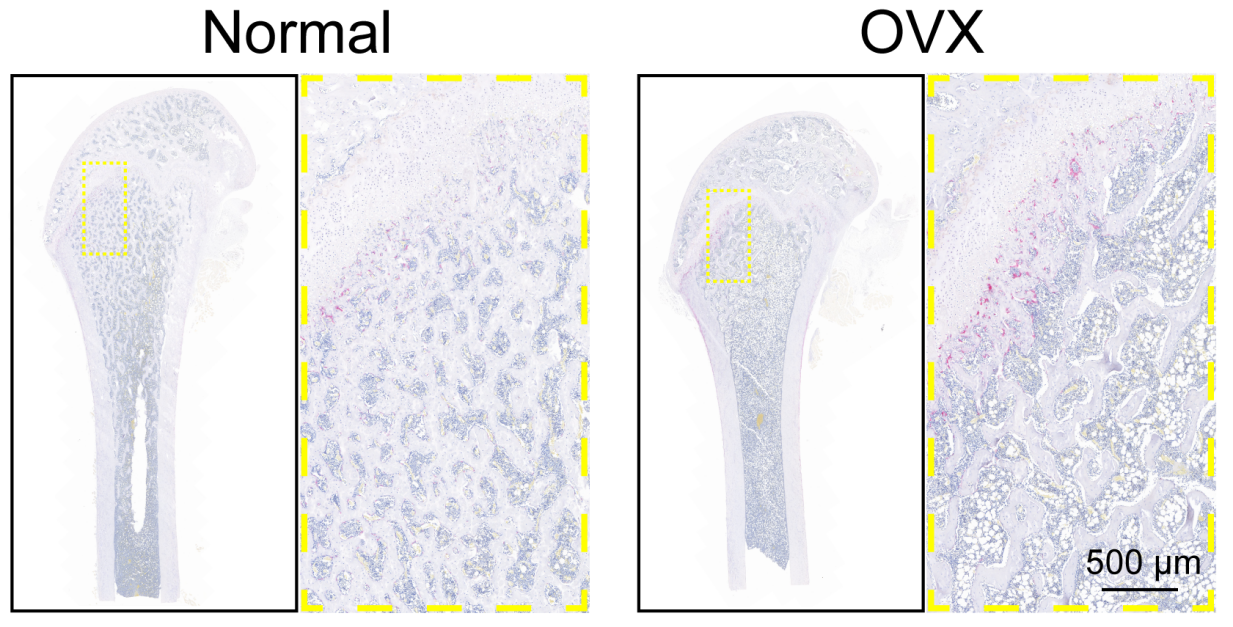


**Figure S16.** TRAP staining of femurs from normal and OVX rats.


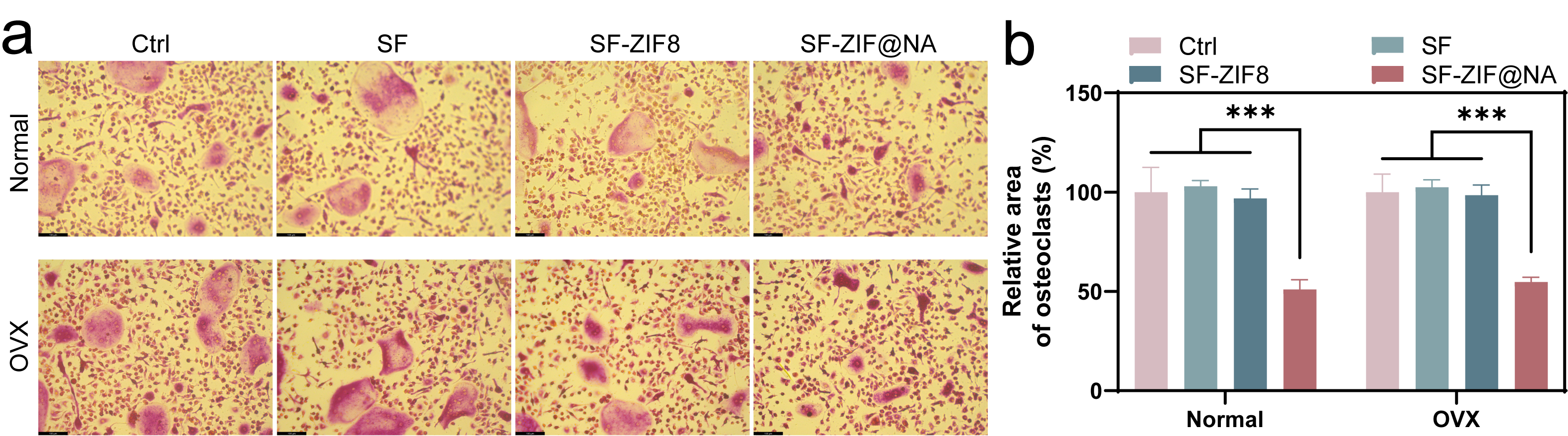


**Figure S17.** (a) TRAP staining images and (b) quantitative analysis of BMMs from Normal and OVX rats after different treatments (scale bar: 100 μm). Data are presented as mean ± SD (n=3, one-way ANOVA). *p <0.05, **p <0.01, ***p <0.001 indicate statistical significance.


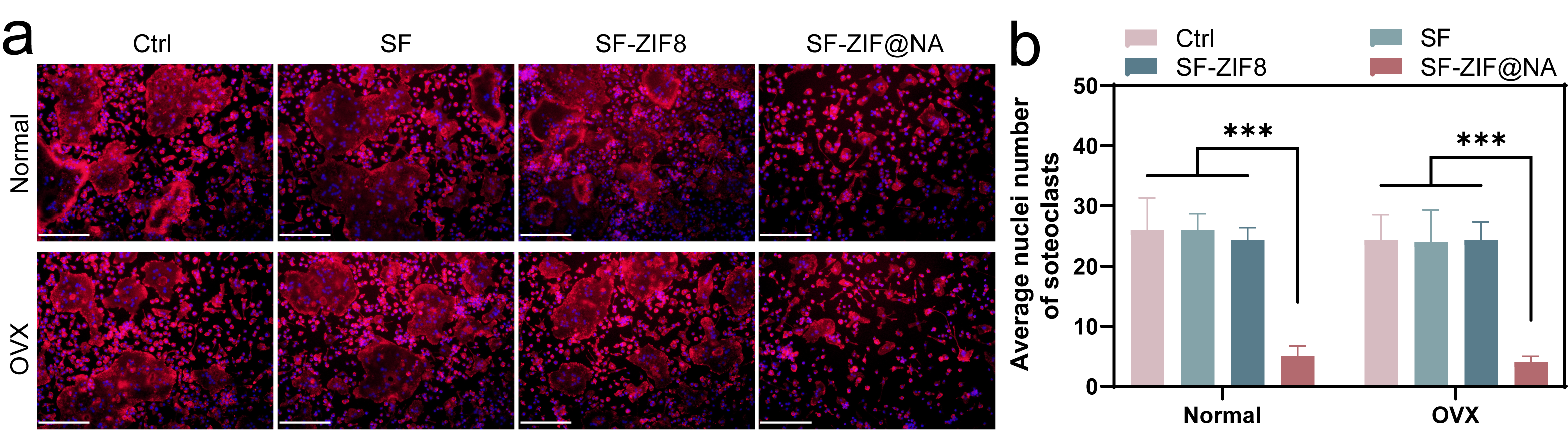


**Figure S18.** (a) The nuclear/F-actin staining and (b) quantitative analysis of BMMs from Normal and OVX rats after different treatments (scale bar: 200 μm). Data are presented as mean ± SD (n=3, one-way ANOVA). *p <0.05, **p <0.01, ***p <0.001 indicate statistical significance.


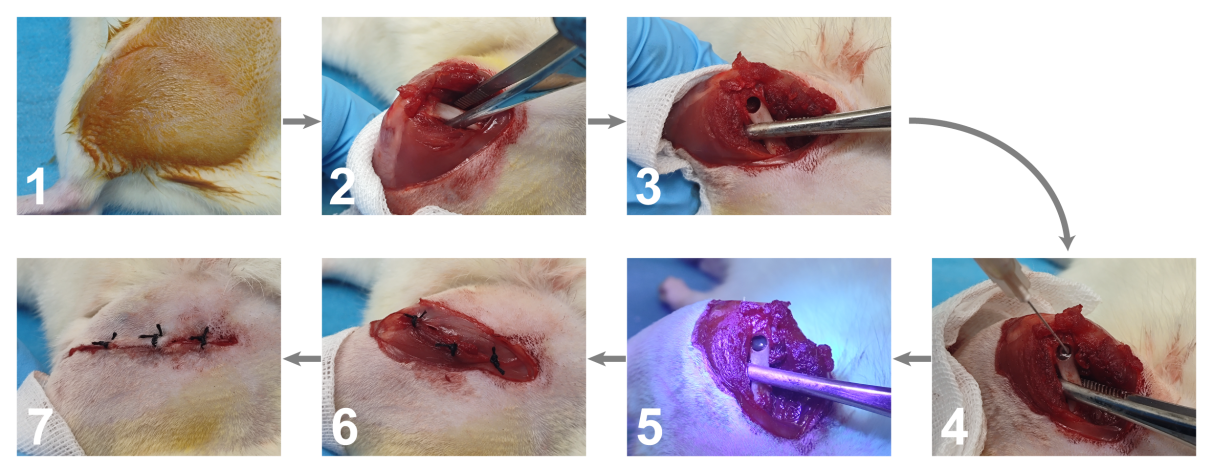


**Figure S19.** Surgical procedure for establishing the femoral defect model and implanting the hydrogel. The surgical operation of implanting was as follows: 1)Disinfection and preparation of the skin; 2)Skin and muscle incision to expose the femur; 3)Creation of a cylindrical defect (diameter: 3 mm, depth: 2.5 mm) at the distal femur; 4)Filling the defect with the hydrogel; 5)Crosslinking the hydrogel using UV light; 6)Suturing the muscle layer; 7)Suturing the skin.


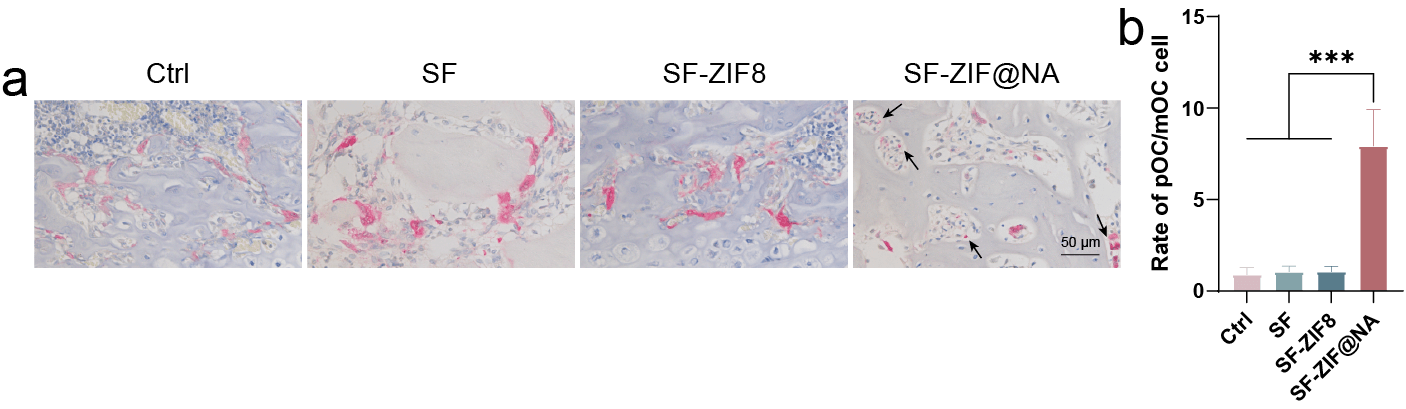


**Figure S20.** (a) TRAP staining and (b) quantitative analysis of femoral defects at 8 weeks post-implantation. Data are presented as mean ± SD (n=3, one-way ANOVA). *p <0.05, **p <0.01, ***p <0.001 indicate statistical significance.


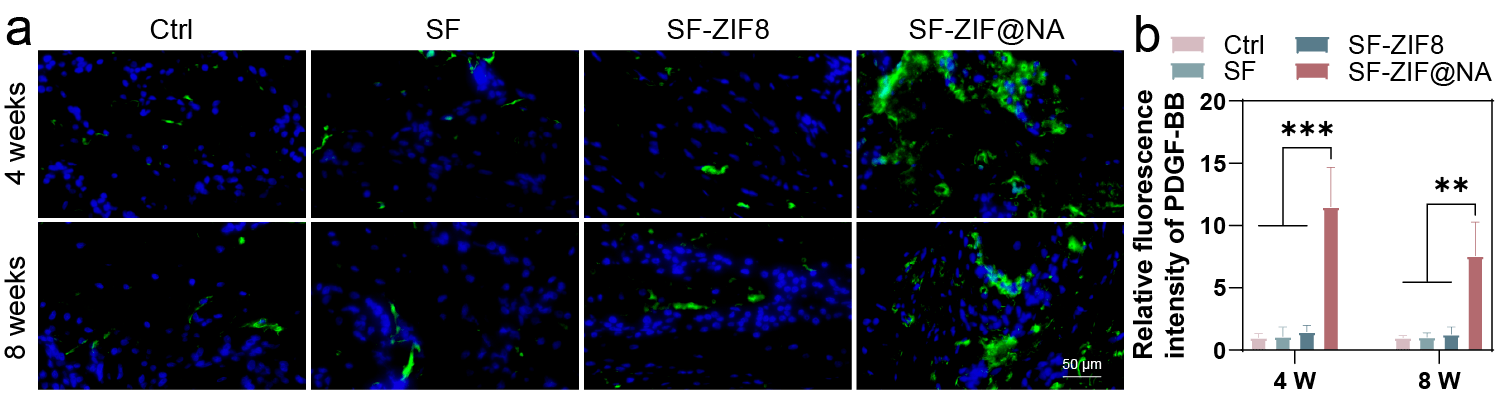


**Figure S21.** (a) PDGF-BB immunofluorescence staining and (b) quantitative analysis of femoral defects at 4 and 8 weeks post-implantation. Data are presented as mean ± SD (n=3, one-way ANOVA). *p <0.05, **p <0.01, ***p <0.001 indicate statistical significance.


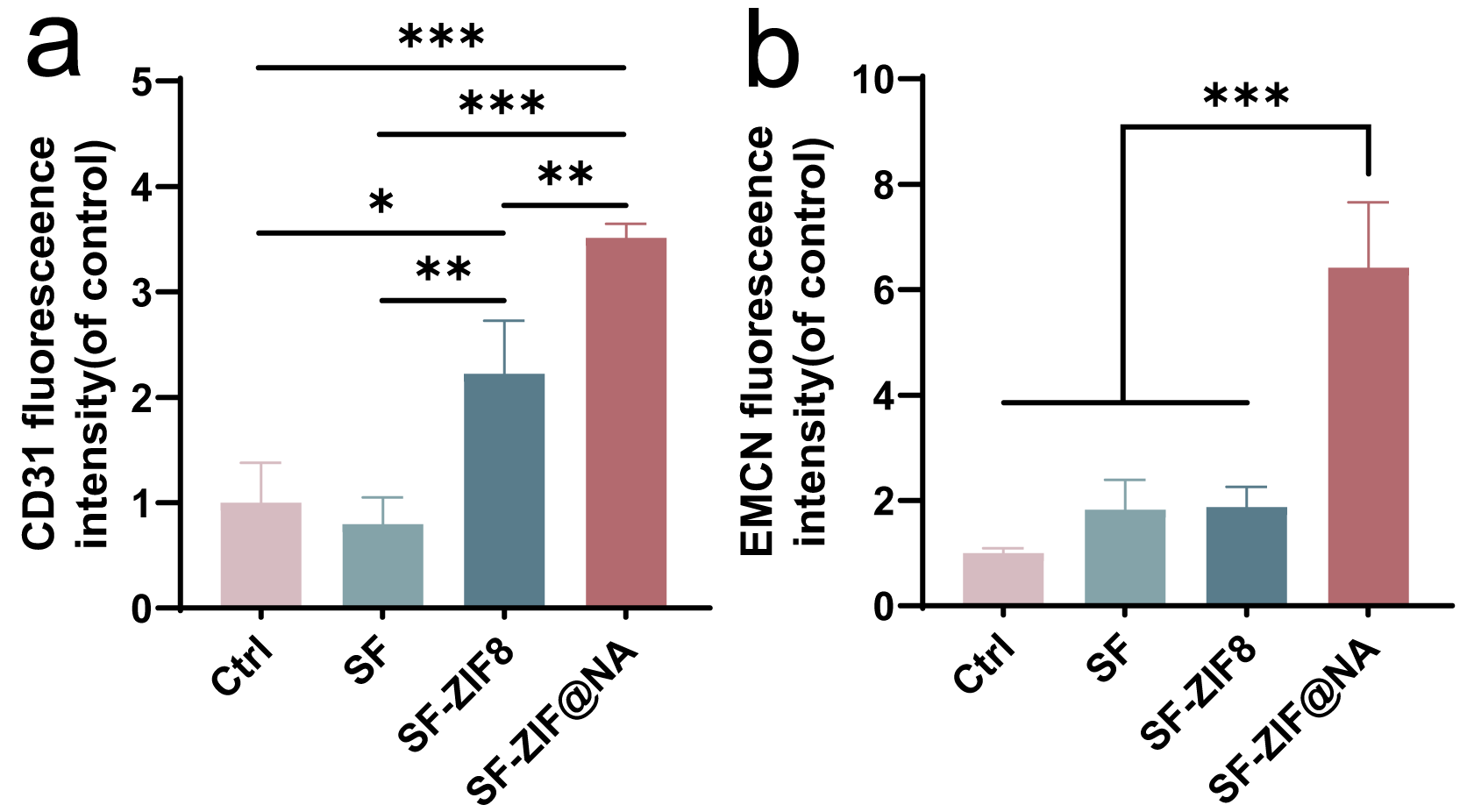


**Figure S22.** Quantitative analysis of (a) CD31 and (b) EMCN staining in femoral defects 8 weeks post-implantation. Data are presented as mean ± SD (n=3, one-way ANOVA). *p <0.05, **p <0.01, ***p <0.001 indicate statistical significance.


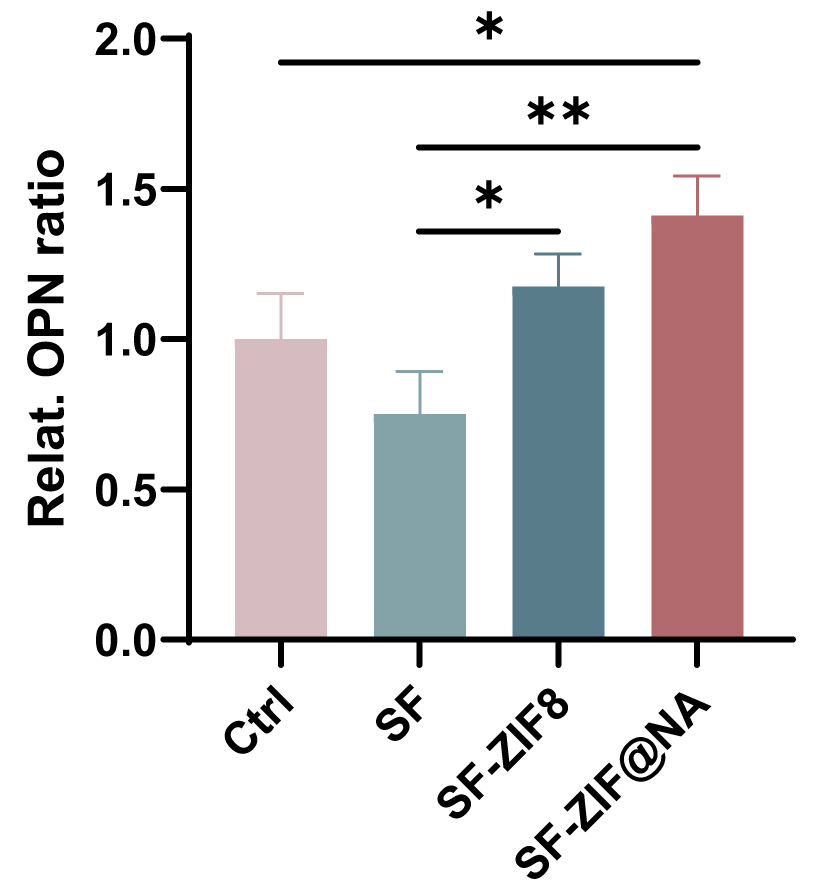


**Figure S23.** Quantitative analysis of the immunohistochemical staining of the osteogenic marker OPN in femoral defects 8 weeks post-implantation. Data are presented as mean ± SD (n=3, one-way ANOVA). *p <0.05, **p <0.01, ***p <0.001 indicate statistical significance.


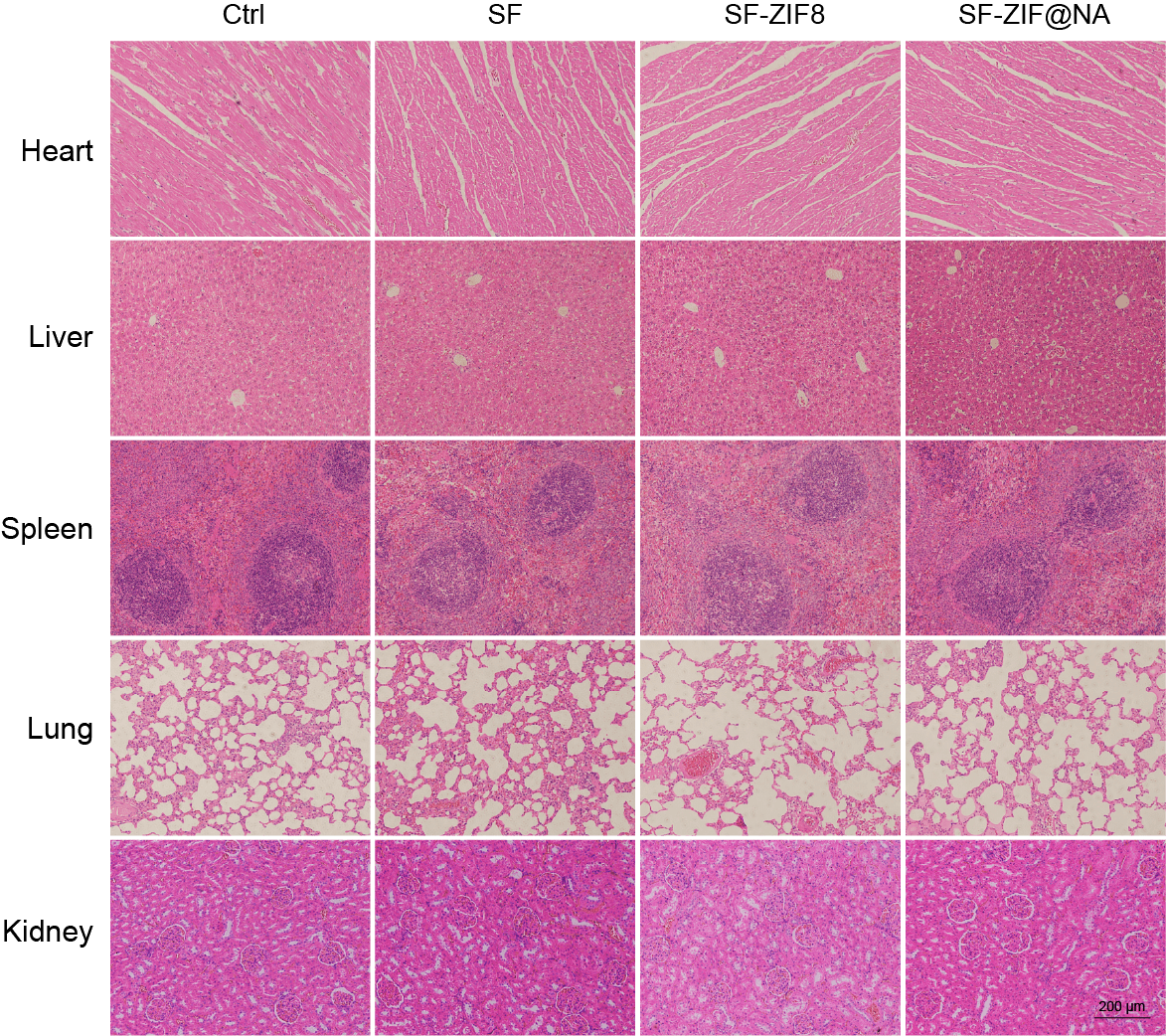


**Figure S24.** HE staining of heart, liver, spleen, lungs, and kidney after 8 weeks of treatment.

**Table S1**. Nanocomposite hydrogels with different ratios.

| Samples | SFMA  (mg/mL) | Nanoparticles  (mg/mL) |
| --- | --- | --- |
| SF | 150 | 0 |
| SF-ZIF@NA | 150 | 0.1 ZIF@NA |
| SF-ZIF@NA | 150 | 0.25 ZIF@NA |
| SF-ZIF@NA | 150 | 0.5 ZIF@NA |
| SF-ZIF@NA | 150 | 1 ZIF@NA |
| SF-ZIF@NA | 150 | 2 ZIF@NA |
| SF-ZIF8 | 150 | 1 ZIF8 |

**Table S2.** Enzyme activity in supernatant before and after synthesis and enzyme loading efficiency.

| Before (OD value) | After (OD value) | Enzyme loading efficiency (%)  $\frac{\text{Before－After}}{\text{Before}}$*100% |
| --- | --- | --- |
| 10903749 | 8390790 | 22.21 |
| 11077096 | 8432955 | 23.37 |
| 11966985 | 8338254 | 32.07 |
| 11315943 ± 570442 | 8387333 ± 47445 | 25.88 ± 5.389 |
